# Asymmetric trichotomous partitioning overcomes dataset limitations in building machine learning models for predicting siRNA efficacy

**DOI:** 10.1016/j.omtn.2023.06.010

**Published:** 2023-06-14

**Authors:** Kathryn R. Monopoli, Dmitry Korkin, Anastasia Khvorova

**Affiliations:** 1 Department of Bioinformatics & Computational Biology, Worcester Polytechnic Institute, Worcester, MA 01609, USA; 2 RNA Therapeutics Institute, University of Massachusetts Chan Medical School, Worcester, MA 01655, USA

**Keywords:** MT: Oligonucleotides: Therapies and Applications, siRNA, RNA interference, machine learning, artificial intelligence, oligonucleotides, oligonucleotide therapeutics, computational model development, chemical modifications, random forest, supervised learning

## Abstract

Chemically modified small interfering RNAs (siRNAs) are promising therapeutics guiding sequence-specific silencing of disease genes. Identifying chemically modified siRNA sequences that effectively silence target genes remains challenging. Such determinations necessitate computational algorithms. Machine learning is a powerful predictive approach for tackling biological problems but typically requires datasets significantly larger than most available siRNA datasets. Here, we describe a framework applying machine learning to a small dataset (356 modified sequences) for siRNA efficacy prediction. To overcome noise and biological limitations in siRNA datasets, we apply a trichotomous, two-threshold, partitioning approach, producing several combinations of classification threshold pairs. We then test the effects of different thresholds on random forest machine learning model performance using a novel evaluation metric accounting for class imbalances. We identify thresholds yielding a model with high predictive power, outperforming a linear model generated from the same data, that was predictive upon experimental evaluation. Using a novel model feature extraction method, we observe target site base importances and base preferences consistent with our current understanding of the siRNA-mediated silencing mechanism, with the random forest providing higher resolution than the linear model. This framework applies to any classification challenge involving small biological datasets, providing an opportunity to develop high-performing design algorithms for oligonucleotide therapies.

## Graphical abstract

**Figure:**
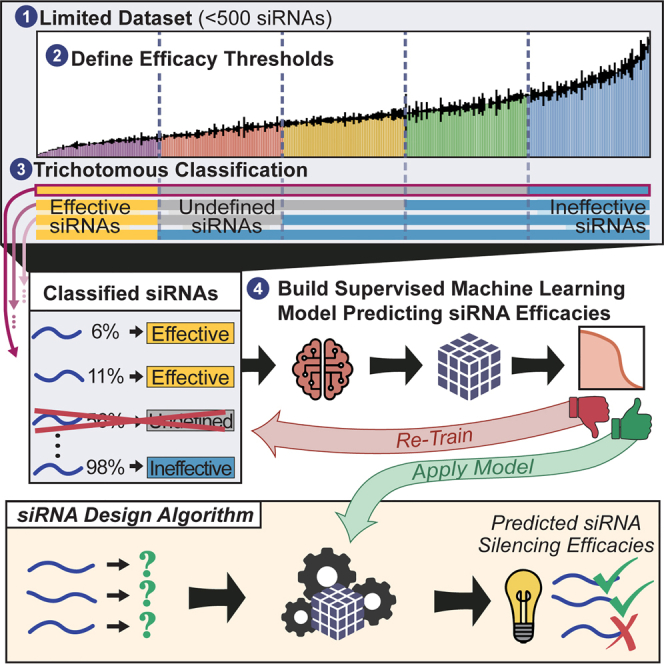

## Teaser

Khvorova and colleagues present a method applying supervised machine learning models to limited-size small interfering RNA datasets. Supervised machine learning models are powerful predictors well suited for identifying potent small interfering RNAs but typically require large datasets. Overcoming this limitation expands application possibilities of machine learning in supporting oligonucleotide therapy development.

## Introduction

**Figure 1:**
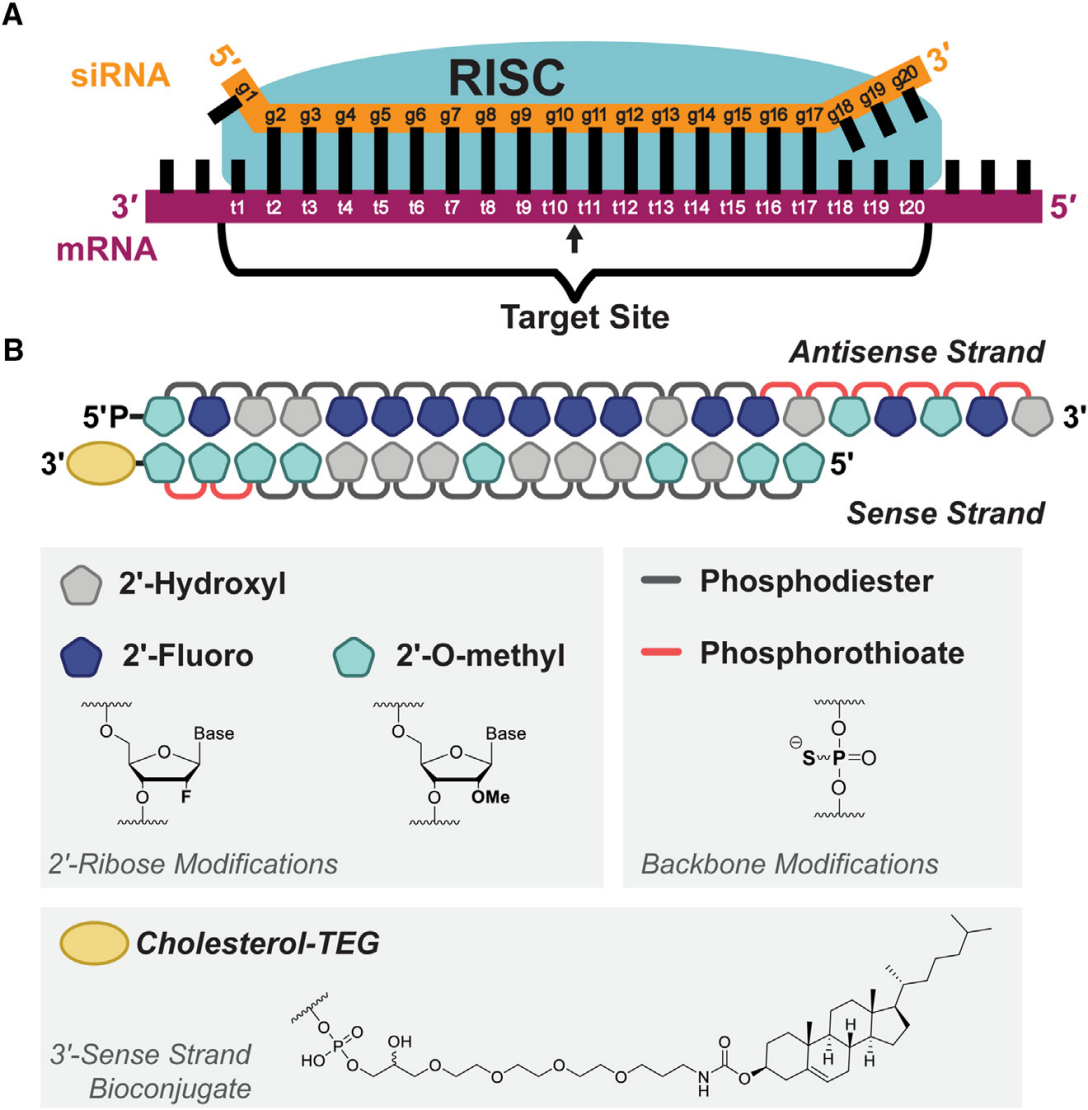
Sequence data used in models and scoring scheme derived from asymmetric, chemically modified siRNAs (A) A 20-nt siRNA, when incorporated into the RNA-induced silencing complex (RISC), binds target mRNA via complementary base-pairing. The siRNA guide strand positions are numbered (g1–g20). The 20-nt target site used for training siRNA design models is indicated, and target positions are numbered (t1–t20). The sequence of this region is used to train all models. The arrow indicates the location of mRNA cleavage by the RISC between positions t10 and t11. (B) Chemical scaffold of asymmetric siRNAs evaluated previously by Shmushkovich et al.^10^ consists of 15-nt sense and 20-nt antisense strands. Cholesterol was conjugated to the 3′ end of the sense strand. The first two 3′ terminal sense strand linkages were phosphorothioated. All sense-strand pyrimidines were 2′-O-methyl modified. The first six 3′ terminal antisense strand linkages were phosphorothioated. All antisense strand pyrimidines were 2′-fluoro modified. The first antisense base from the 5′ end was fixed to 2′-O-methyl uridine.

Small interfering RNA (siRNA) drugs guide potent and specific silencing of disease-related genes. siRNAs direct gene silencing by loading into the RNA-induced silencing complex (RISC) and binding the target site of an mRNA via complementary base pairing (Figure 1A).^1^^,^^2^^,^^3^ The RISC then cleaves the target transcript, triggering mRNA degradation.^1^^,^^2^^,^^3^^,^^4^ With the recent US Food and Drug Administration (FDA) approval of five siRNA drugs (patisiran, givosiran, lumasiran, inclisiran, and vutrisiran) and many other siRNAs in late-stage clinical trials, siRNAs have become one of the most promising drug modalities.^5^^,^^6^^,^^7^^,^^8^^,^^9^

Despite siRNA sequence being a predictor of efficacy (i.e., degree of target gene silencing),^10^ identification of siRNA target sites that effectively reduce target gene expression *in vivo* remains a key bottleneck in siRNA drug development. Therapeutic siRNAs must be fully chemically modified to increase stability and bioavailability *in vivo*,^11^^,^^12^^,^^13^ and tolerability of chemical modifications is a primary factor limiting siRNA efficacy. Although publicly available algorithms can accurately determine effective native siRNAs, their prediction accuracy is poor when applied to chemically modified compounds.^10^ Moreover, early “first-generation” siRNA design algorithms often employed simple learning architectures, such as linear models, which cannot describe complex sequence relationships that might underlie siRNA efficacy.^14^^,^^15^^,^^16^^,^^17^^,^^18^

Machine learning (ML) approaches that leverage non-linear models are highly powerful in fitting complex patterns in the data^19^^,^^20^^,^^21^^,^^22^ and can be applied to build algorithms of superior predictive power. Supervised ML methods focused on classification (e.g., effective versus ineffective) achieve this by training a model on a labeled dataset, with each data point assigned to a specific class label. However, many supervised ML methods require hundreds or thousands of labeled data points to build a model that accurately classifies the previously unlabeled data^19^^,^^20^^,^^21^^,^^22^ and, thus, are difficult to apply to modified siRNA efficacy datasets. Most data are industry generated and, thus, proprietary, with the cost of synthesis and screening further limiting data availability to the research community. Studies applying ML to non-modified siRNA data have increased dataset size by combining heterogeneous siRNA data (generated from structurally and chemically different compounds using different assays/conditions).^23^^,^^24^^,^^25^ However, applying ML to heterogeneous data can generate errors, which reduces prediction accuracy.

Here, we apply a supervised ML approach to a previously published small chemically modified siRNA efficacy dataset (n = 356) by using the data themselves to inform the classification process.^10^ Introducing a two-threshold (or trichotomous) model combined with a systematic assessment of a range of classification thresholds overcomes bias introduced by defining a single *ad hoc* threshold. This trichotomous scheme enables optimization of threshold pair selection to significantly reduce noise generated from using small siRNA datasets where large variability in signal is common.^10^ The resulting ML model showed high predictive power and outperformed a linear classification model built from the same data. To assess model validity and propose biological mechanisms underlying model results, we evaluated features dictating model prediction using a novel method for extracting sequence position base weights. In contrast to the previous approaches, this feature extraction method employs an evaluation-centered protocol whose application method is completely agnostic to model type and can be computed quickly in the context of the large number of position base features present in sequence data.^26^^,^^27^^,^^28^ We experimentally assessed our framework in the context of siRNA design by applying the ML model to select a panel of siRNAs targeting four human genes. Of the siRNAs predicted by the model to be the topmost effective silencers, 7 of 10 showed potent silencing (<22% reporter expression remaining) when evaluated in cells with a dual-luciferase reporter assay. The two-threshold framework presented here can be applied to any noisy biological dataset to build powerful ML models and is designed to perform well even on small datasets. Such a framework unlocks the fully utility of siRNA datasets, which are typically small and noisy, to better understand siRNA mechanisms and design next-generation nucleic acid therapeutics.

## Results

### siRNA efficacy dataset and two-threshold class annotation

A chemically modified siRNA efficacy dataset consisting of 356 target sequences was used for ML model training.^10^ The dataset comprises compounds targeting 17 genes, with an average of 15 siRNAs per gene. Sequences were designed with minimal constraints, mostly limited to favoring low GC content. All siRNAs were designed with the same asymmetrical chemical modification pattern (Figure 1B) to enhance cellular uptake and stability, which enhances potency.^13^^,^^29^^,^^30^^,^^31^^,^^32^^,^^33^ The asymmetrical pattern consists of a 15-nt sense and 20-nt antisense strand. On the sense strand, all pyrimidines were 2′-O-methyl modified, and the first two 3′ terminal linkages were phosphorothioated. On the antisense strand, all pyrimidines were 2′-fluoro modified, the first six 3′ terminal linkages were phosphorothioated, and the first base from the 5′ end was fixed to a 2′-O-methyl uridine. To remove unmodified ribose stretches, purine modifications were added to both sense and antisense strands. The asymmetric structure and 3′ modifications on the sense strand ensure proper strand loading into the RISC.^33^ Conjugation of cholesterol to the 3′ end of the sense strand enhances delivery of these siRNAs into cells.^34^ There are no other uniform (same assay, experimental setup, and modification pattern), diverse (different genes) modified siRNA datasets publicly available for analysis, but the intention of the presented framework is applicable to any future datasets.

This chemical modification pattern has a profound impact on siRNA efficacy because the functional asymmetry required for proper strand loading is introduced through these modifications rather than sequence. In most existing siRNA efficacy algorithms, thermodynamic bias is a primary predictor; thus, these algorithms are not predictive on modified siRNA,^14^^,^^17^^,^^35^^,^^36^ which we have confirmed previously.^10^

The 20-nt target site sequence for each siRNA was used as a training set for the supervised ML model. Base weights at each position of a target site (Figure 1A) were used as features—4 bases × 20 nt positions = 80 position base features—to encode representation of each data point (see materials and methods for feature parametrization). Including other features of siRNA targeting (i.e., target mRNA structure, target abundance, mRNA sequence flanking the target site) could potentially improve model performance; however, for simplicity, we focus on target site sequence because it is a key predictor of siRNA efficacy. Importantly, modified siRNAs, being highly structured small sequences, are not impacted by steric effects, particularly in the context of identical modification patterns, as is the case in the dataset used here.^37^^,^^38^

**Figure 2:**
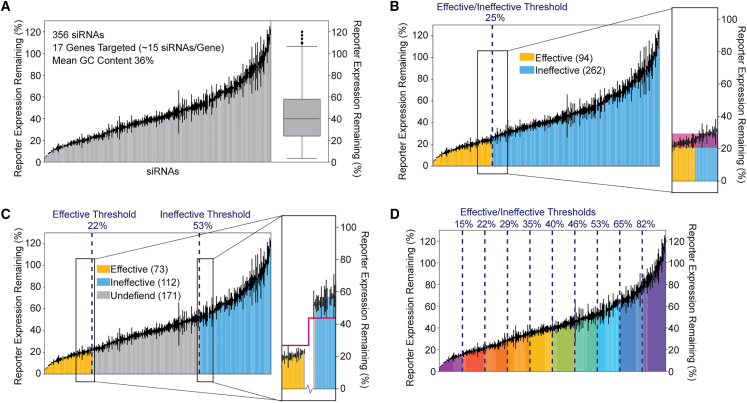
Noisy data and intermediate values challenge siRNA classification (A) Gene silencing efficacy for 356 chemically modified siRNAs evaluated previously by Shmushkovich et al.,^10^ targeting 17 different genes (∼15 siRNAs/gene) in HeLa cells using a dual-luciferase assay normalized to nontreated cells.^10^ Each bar represents the efficacy of a single siRNA sequence averaged over three independent measurements, with error bars depicting the standard deviation. The box-and-whisker plot depicts the distribution of siRNA efficacies across the entire dataset. (B and C) Data in (A) classified as effective (yellow), ineffective (blue), or undefined (gray) by the thresholds indicated (dark blue dotted lines; threshold reporter expression percentage indicated at the top). The number of siRNAs in each class is indicated in parentheses. The inset shows regions around thresholds in greater detail. Shaded maroon boxes indicate regions with overlapping noise in the effective and ineffective classes (from maximal standard deviation value in the effective class to minimal standard deviation value in the ineffective class). Maroon bars indicate regions without overlap between effective and ineffective classes. (D) Data in (A) with all nine evenly spaced thresholds used in the evaluation (dark blue dotted lines; threshold reporter expression remaining percentage indicated at top). Spans between thresholds defined 35–36 siRNAs. Threshold pairs contained all possible combinations of nonoverlapping effective/ineffective thresholds, resulting in 45 possible combinations. Effective classes contained all siRNA sequences less than or equal to the threshold. Ineffective classes contained all siRNA sequences greater than the threshold.

siRNA efficacy was determined by a dual-luciferase reporter assay^10^ and defined as reporter expression in cells treated with siRNA as a percentage of reporter expression in untreated cells. siRNA efficacies ranged from 4%–120% reporter expression, with a mean and median of 44% and 40%, respectively (Figure 2A). The luciferase reporter allows unification of the siRNA dataset by using a single experimental measure of efficacy. The average percent error was 3%, and individual siRNA efficacy values varied up to 16% (Figure 2A).

Consistent with other siRNA efficacy datasets, distribution of the data did not provide a clear threshold for classification (Figure 2A). A biologically reasonable threshold of 25% reporter expression (Figure 2B) would define 94 effective siRNAs. However, data points at both sides of the threshold have a large overlap in error bars (Figure 2B, inset). This noise makes data points around the threshold indistinguishable—a point directly to the right of the threshold is no different from a point to the left of it. Thus, this classification will result in a subset of sequences with biologically equivalent efficacies distributed to different classes.

To overcome this issue, we applied a non-conventional trichotomous grouping method that uses two independently selected thresholds: one defined effective siRNAs (h_1_), and the other defined ineffective siRNAs (h_2_). siRNAs with reporter expression values of less than or equal to the selected h_1_ threshold were labeled “effective,” while those with values greater than the selected h_2_ threshold were labeled “ineffective.” All siRNAs lying between these thresholds were classified as “undefined” and excluded from model development (Figure 2C). This thresholding scheme results in two clearly distinct groups with no “noise overlap” (Figure 2C, inset).

The trichotomous partitioning method has been applied previously;^10^ however, the systematic evaluation of differing thresholds and their impact on model performance has not been performed. To optimize determination of effective and ineffective siRNA threshold values, different pairs of h_1_ and h_2_ efficacy thresholds were considered for testing from a range of nine equally distributed reporter expressions ranging from 15%–82% (Figure 2D). All permutations of effective and ineffective threshold pairs were systematically evaluated with the constraint that h_1_ ≤ h_2_ to exclude threshold combinations that would classify the same siRNA(s) into both effective and ineffective classes (e.g., h_1_ ≤ 15%, h_2_ >15% was considered but h_1_ ≤ 35% and h_2_ >22% was not). In the cases where h_1_ = h_2_, all siRNAs were classified as either effective or ineffective, and no undefined siRNAs were classified. Thus, a total of 45 threshold combinations were considered. The size of the dataset used for model building was affected by threshold selection; models built with the most stringent threshold (h_1_ ≤ 15% and h_2_ > 82% – hereafter written as 15/82) were built using data from the fewest siRNAs, while models built using identical effective and ineffective thresholds use the whole dataset (e.g., 15/15, 22/22, etc.).

### Pipeline for classification model development

**Figure 3:**
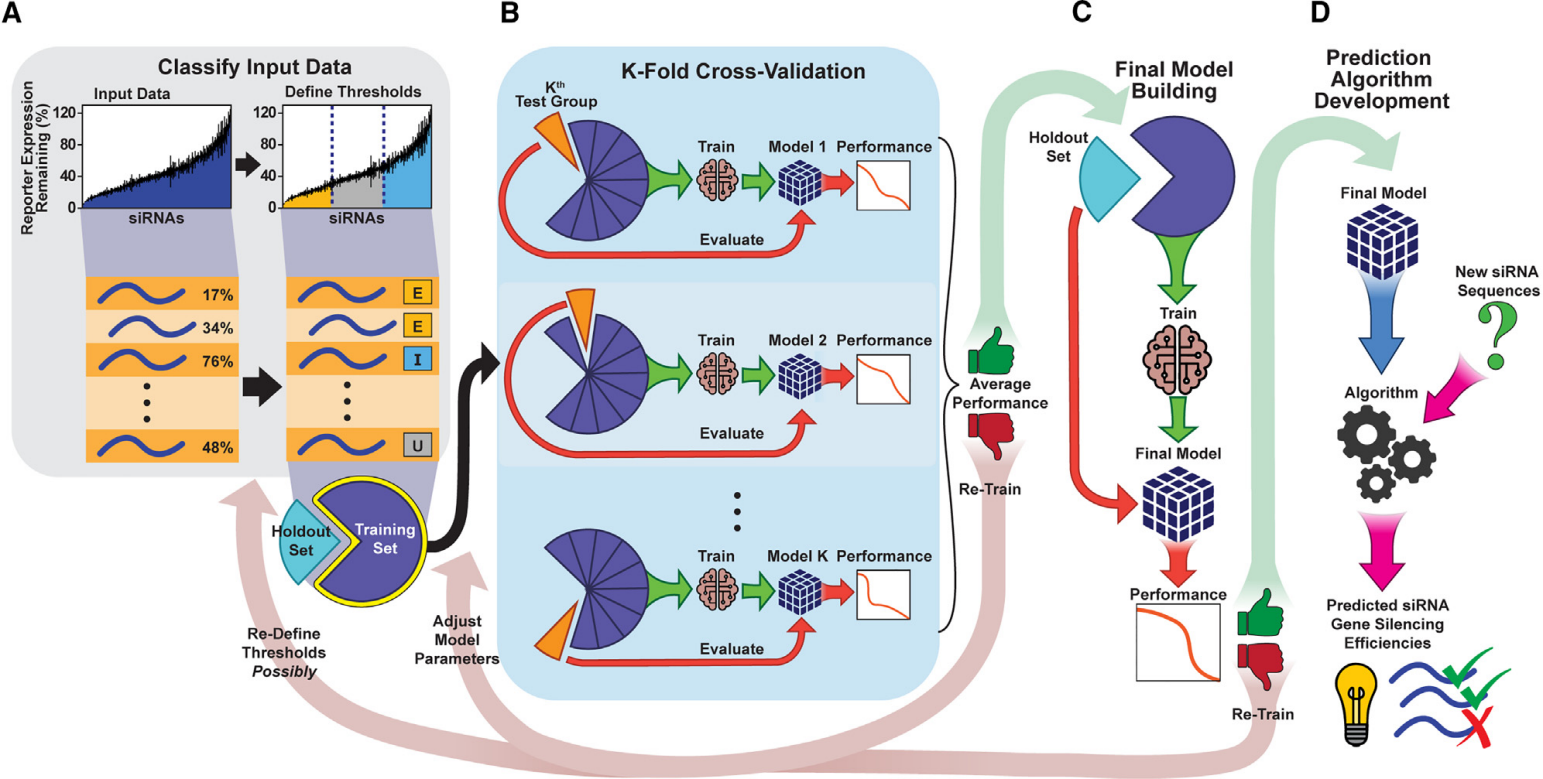
Schematic for training the supervised ML model to produce an efficacy prediction algorithm for a single threshold combination (A) Input siRNA sequences with experimentally determined gene silencing efficacies are classified using predefined thresholds (dark blue dotted lines) into three groups: effective (E), ineffective (I), and undefined (U). Classified data are partitioned randomly into holdout (25% of data) and training (75% of data) sets. (B) Training data are split into K (10) subsets of equal size. The Kth test set consisting of 1/10th (one subset) of the training data is held out (orange pie slice), and a model is trained on the remaining 9/10th (nine subsets) of the training data. Model performance is evaluated using the Kth test set. If the average performance of all K models is acceptable, then final model building can proceed; otherwise the model must be re-trained. (C) Using the full training set, the final model is built, and its performance is evaluated on the holdout set. If the performance is acceptable, then prediction algorithm development can proceed using the model; otherwise the model must be re-trained. (D) The final model is used to build an algorithm to predict gene silencing efficacies of siRNA sequences whose efficacies have not been experimentally determined.

We evaluated the impact of all 45 threshold combinations on model performance using the pipeline in Figure 3. For each threshold pair (Figure 3A), a supervised ML model employing random forest (RF) classification was built. RF was selected because this ML model type is known to achieve a learning plateau in the fastest way, requiring the fewest number of training examples among all nonlinear ML methods.^39^ RF models use decision trees to partition data by their features to classify the data. In our analysis, the RF models partition data by target site position base features to classify siRNAs as positive (i.e., effective) or negative (i.e., ineffective). RF performs well on data with a large number of features, and the trees and branching structure of RF have the potential to capture complex interactions (e.g., sequence motifs) that a simpler linear model cannot.^22^

For model development and independent validation, the dataset was partitioned into a training set and holdout set, consisting of 75% and 25% of the data, respectively (Figure 3A). These partition proportions were selected because they showed the greatest overall model performance (Figure S1). The data were split to ensure equal distribution of effective and ineffective siRNAs (per selected threshold pair for that model) into the training and holdout sets to minimize biases and optimize model development (see materials and methods for the assessment protocol).

Because the training and holdout sets inherently have different characteristics, bias can be introduced into the model during partitioning. This is particularly true for small, diverse datasets because anomalies existing in only a few data points (as few as five siRNAs) can cause a model to underperform. This bias is minimized using K-fold cross-validation, an iterative process in which the training set is randomly partitioned into K groups of equal size, and then K rounds of model building are performed using K-1 groups in training and 1 group in testing.^40^ The testing group is then used for model evaluation (Figure 3B). For siRNA prediction models, K was set to 10, a typical number for a dataset of this size, and the cross-validation process was repeated a total of 10 times so that each partition served as the testing group once. The average performance of the K models was then analyzed (see results for the novel scoring metric for model evaluation). Default values of the standard parameters of an RF model, the depth of the tree and the number of trees, were chosen because altering these parameters did not impact model performance (data not shown). Following K-fold cross-validation, final model training was performed, with the entire training set evaluated on the holdout set (Figure 3C). Because the holdout set was not involved in K-fold cross-validation, model performance on this set is a strong indicator of model generalization (i.e., performance on future unseen data). The final model is then used to build an algorithm to predict effective and ineffective siRNAs (Figure 3D).

### A novel scoring metric, area under the precision-recall curve adjusted (AUCPR_adj_), for model evaluation across two-threshold combinations

Accuracy, a popular model performance metric that measures correct versus total predictions, is misleading in the context of large class imbalances.^41^ This is of particular concern for fully modified siRNA efficacy datasets, like the one used here, in which there are many more ineffective siRNAs than effective siRNAs for a target transcript. Another popular model performance metric is the area under the receiver operating characteristic (ROC) curve, which plots the true positive rate (also called recall) against the false positive rate.^42^ The area under the ROC curve (AUC) can be used to quantify this metric. However, like accuracy, ROC curves (and their corresponding AUCs) do not account for imbalanced data, overestimating model performance in datasets dominated by positively classified values (i.e., permissive efficacy threshold).

The precision-recall curve is a better metric for siRNA design models.^43^ Recall (Equation 1) depicts the fraction of siRNAs correctly predicted as effective with respect to all effective siRNAs in the dataset.^43^ A model producing a large number of false negatives (effective siRNAs classified as ineffective) will have low recall. Precision (Equation 2) is the fraction of siRNAs correctly predicted as effective with respect to all siRNAs predicted to be effective.^43^ A model producing a large number of false positives (ineffective siRNA classified as effective) will have low precision. When combined, recall and precision consider false negatives and false positives to capture both class types, overcoming class imbalance issues in model evaluation.

Many classification models are inherently probabilistic.^44^ When performing a prediction, an RF classification model puts out a confidence score ranging between 0 and 1.^22^ Confidence scores closer to 1.0 indicate a greater probability of a particular value being predicted by the model to be positive (effective).^22^ A confidence margin is applied to the score, producing a binary classification.^44^ A margin of 0.5 is typically selected initially by default for a model.^44^ Tuning this margin can sometimes provide greater predictive power of a model but requires a large independent dataset to optimize the margin; thus, in this study, we maintain the default 0.5 confidence margin.^45^^,^^46^ The precision-recall curve is constructed by computing precision and recall values across the range of confidence margins (from 0–1).

(Equation 1) $$Recall=True\;Positive\;Rate=\frac{True\;Positives}{True\;Positives+False\;Negatives}$$

(Equation 2) $$Precision=\frac{True\;Positives}{True\;Positives+False\;Positives}$$

The goal of siRNA design is to identify an siRNA sequence that effectively silences a target gene. Typically, only a single effective siRNA needs to be identified to achieve this silencing. Thus, a strong model for this application does not need to identify all possible effective siRNA sequences, but the sequences it does identify should have a high probability of being effective. Such a model will prioritize high precision (majority of siRNAs classified as effective are effective) over high recall (identifying all possible effective siRNAs). A strong model also does not need to excel at identifying ineffective siRNA sequences, thus identifying some false negatives (siRNAs that are truly effective but classified by the model as ineffective) is acceptable.

**Figure 4:**
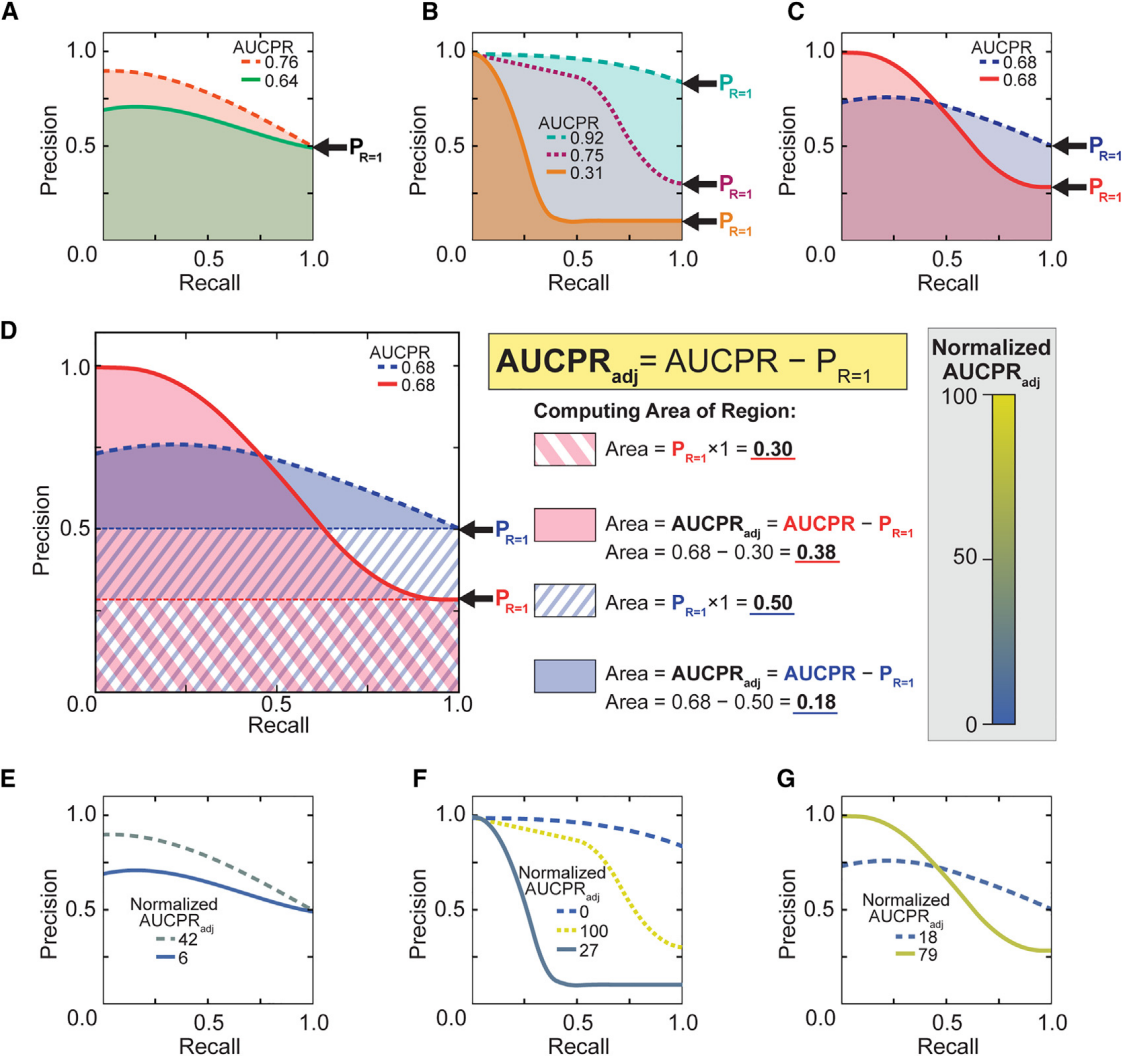
AUCPR_adj_ overcomes challenges of model evaluation (A) Precision-recall curves for two different models developed using the same threshold values. Curves depict model performance for a better-performing model (orange dashed curve) and a worse-performing model (green curve). Areas under the precision-recall curve (AUCPRs), represented by shaded regions, are indicated at the top. The arrow identifies precision when recall equals one (P_R=1_). (B) Precision-recall curves of a model with no discriminatory ability, with AUCPR overestimating performance (teal dashed curve), an underfit model with AUCPR underestimating performance (gold line), and a top-performing model (purple dashed curve). Arrows identify P_R=1_ values for the curve of the corresponding color. (C) Precision-recall curves for models developed with different E thresholds: a more stringent threshold (red curve) and a more permissive threshold (blue dashed curve), resulting in curves with identical AUCPRs. (D) Same data as in (C), depicting AUCPR_adj_ (red- and blue-shaded regions) derivation by subtracting the area defined by the P_R=1_ (red/white- and blue/white-striped regions) from the corresponding curve’s (red or blue) AUCPR. The general formula for computing AUCPR_adj_ is described (yellow box). Detailed adjusted AUCPR (AUCPR_adj_) derivations for the blue and red curves are provided (center). The color bar represents the scheme used throughout this manuscript for color-coding curves by AUCPR_adj_ values normalized between 0 and 100. (E–G) Same curves as in (A)–(C), respectively; colored by normalized AUCPR_adj_.

The AUCPR converts the precision-recall curve to a single numeric value.^43^ A higher AUCPR usually indicates a better-performing model (Figure 4A). Unfortunately, in the context of vastly different h_1_ thresholds, the AUCPRs of different curves are not comparable because changing h_1_ affects the precision when recall equals 1 (P_R=1_), causing more permissive h_1_ thresholds to automatically generate a higher AUCPR (Figure 4B, blue vs. gold curves) and allowing two models with different thresholds and performance to potentially produce identical AUCPRs (Figure 4C). To overcome this, we adjusted the AUCPR to the P_R=1_ by subtracting the area defined by precision at maximum recall, creating a metric we defined as AUCPR_adj_ (Figure 4D; Equation 3). This adjustment maintains proper performance assessment of models built with the same h_1_ threshold (Figure 4E), corrects for poor (low AUCPR_adj_) assessment of underfit models and models with no discriminatory ability (Figure 4F), and distinguishes between models with otherwise identical AUCPRs (Figure 4G). To simplify comparison between different models, we further normalize AUCPR_adj_ to a 1-to-100 scale, creating a standalone performance metric (Figure 4D, color scale bar).

(Equation 3) $${AUCPR}_{adj}=AUCPR-P_{R=1}$$

In addition, contingency tables, which quantify true positive, false positive, true negative, and false negative groups, are used to examine the source of poor performance (Figures S2 and S3). By depicting binary classification at a single confidence margin, contingency tables show the discrete output of a model as it would be applied in prediction (i.e., siRNA design). Here these tables are defined at the 0.5 confidence margin, which is typical.^44^ Contingency tables serve to complement precision-recall curves where discrete classification outputs cannot be determined directly.

### Performance of the RF model for siRNA prediction is highly affected by classification thresholds

**Figure 5:**
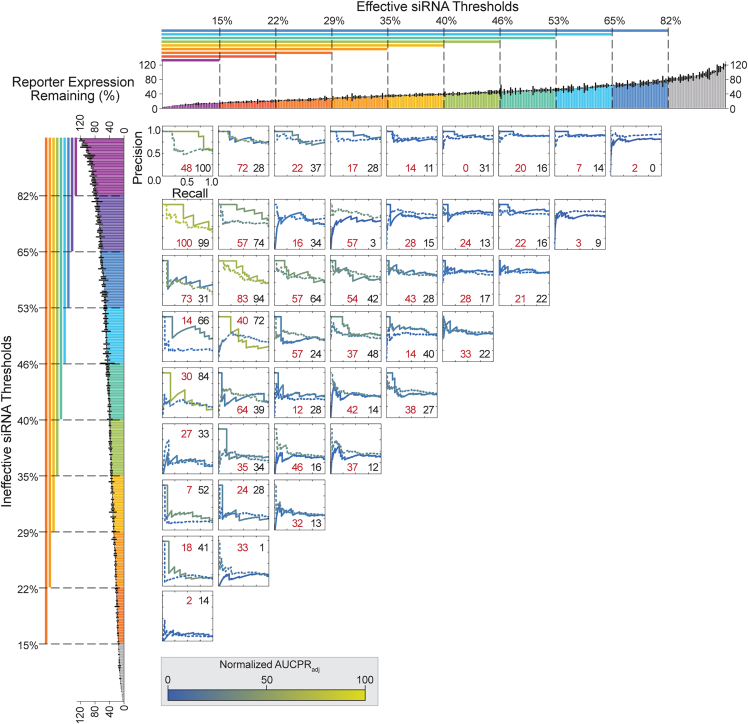
Model performance per classification threshold Precision-recall curves depicting model performance during evaluation on the holdout set (solid lines) and K-fold cross-validation (dotted lines). For holdout set evaluation, RF classifiers were trained on the entire training set and evaluated on the holdout set. For K-fold cross-validation (with K = 10), RF classifiers were trained on 9/10th training subsets and evaluated on the corresponding 1/10th test subsets, and precision-recall curve values were averaged over 10 rounds of model building. Each plot represents the performance of models trained using different E and I siRNA threshold pairs. One can use AUCPR_adj_ to evaluate model performance. Curves are colored by AUCPR_adj_, which were normalized within each evaluation step (either K-fold cross-validation or holdout set evaluation). The color bar depicts performance by normalized AUCPR_adj_. AUCPR_adj_ values are indicated at the bottom right of each curve K-fold cross-validation (red) or holdout set evaluation (black). Bar plots at the top and left depict all siRNA target expression data (as in Figure 2D), colored by E (top) or I (left) thresholds. Precision-recall curves are aligned to these bar plots to indicate the E and I thresholds used for training of the corresponding curve’s model. Thresholds are inclusive of all data with expression values less than (for E thresholds) or greater than (for I thresholds) the threshold expression percentage. Data used to compute normalized AUCPR_adj_ values can be found in Tables S1 and S2.

Using AUCPR_adj_, we found averaged K-fold cross-validation model performance and final model performance on the holdout dataset (Figure 5; Tables S1 and S2) to be generally similar. There was an overall trend of higher performance for models built with the most stringent threshold pairs (small h_1_/large h_2_; Figure 5, top left curves), while models built with all other threshold combinations performed poorly (Figure 5, bottom left, top right, and center curves).

Top-performing models, defined by a high AUCPR_adj_, utilized threshold pairs that (1) reflect biologically reasonable definitions of siRNA efficacy (<30% reporter expression) and (2) exclude moderate-efficacy siRNA, which might be misclassified and introduce noise. The resulting models have the greatest power to distinguish effective and ineffective siRNAs but come at the cost of excluding a larger amount of data from training. Note the term “top performing” is used here as a comparison with other models evaluated in this study only. We seek in this study to demonstrate the utility of the trichotomous partitioning method and present it within a simplified framework; however, further parameter tuning is likely to produce models with greater predictive power. We do not intend to indicate that these models outperform existing, highly tuned siRNA prediction models.

In K-fold cross-validation, the top-performing threshold pairs (h_1_/h_2_) were 15/65, 15/53, 22/82, and 22/53 (Figure 5). The most stringent threshold pair, 15/82, did not perform well, likely because of the smaller dataset used. In the final model evaluation, AUCPR_adj_ identified 15/82, 15/65, 15/40, 22/65, 22/53, and 22/46 as top-performing threshold pairs (Figure 5). The strong performance of the 15/40 threshold pair, which allows greater inclusion of moderate-efficacy siRNAs, was driven by the identification of true negatives (Figure S3, contingency table). In fact, the model did not identify any true positives, suggesting that the model would not likely perform well for effective siRNA identification. This exemplifies the challenges of model building with a limited dataset, where thresholding can further reduce the evaluation set size (to as few as 17 siRNAs in this assessment) and demonstrates that no single evaluation metric alone is ideal. Considering multiple metrics—in this case, AUCPR_adj_ and the contingency table—is critical for evaluating final model performance.

When evaluating contingency tables, the application of the model is important to consider. The threshold pair 22/53 produced a top-performing model based on AUCPR_adj_; however, the resulting contingency tables show only two siRNAs identified as true positives along with many false negatives (Figure S3). Critical, however, is that no false positives were identified by this model, which, in the context of siRNA design, are highly costly. The large number of false negatives is acceptable because, for siRNA efficacy prediction, the model does not need to excel at identification of negative values. These results further highlight the challenge presented in evaluating models with such a small dataset and further highlight the value of considering multiple metrics (in this case including AUCPR_adj_) in model evaluation.

AUCPR_adj_ successfully identified three categories of poorly performing models. The first category of models utilized moderately effective and ineffective threshold pairs (Figure 5, curves in second through fourth rows from the top and second through fourth columns from the left). Corresponding contingency tables (Figures S2 and S3, center tables) show that the models falsely classify many ineffective siRNAs as effective. This poor performance is likely due to the models including moderate-efficacy siRNAs. The two remaining categories were highly underfit models or those with no discriminatory ability. Underfit models are identified by their low AUCPR and P_R=1_ and were built from threshold pairs producing a larger number of ineffective siRNAs (h_2_ = 29%, 22%, or 15%) (Figure 5, bottom left curves). Models with no discriminatory ability are built from threshold pairs classifying the majority of siRNAs as effective (h_1_ = 53%, 65%, or 82%) (Figure 5, top right curves). Such models have a high AUCPR with a high P_R=1_. This poor performance is likely due to overly permissive effective thresholds mislabeling ineffective siRNAs as effective.

Some threshold pairs produced models that performed notably worse on the holdout set than they did in cross-validation (22/65, 15/46, 22/46, and 15/40) (Figure 5). This is likely due to inherent differences between the holdout and training datasets that were not captured in the model during training and reflects the small dataset size. These discrepancies are not due to unequal representation of effective and ineffective siRNAs in the holdout versus test groups in K-fold cross-validation (Figures S2 and S3); equal representation of effective and ineffective siRNAs was maintained during partitioning of training and holdout sets as well as during k-fold partitioning (materials and methods).

Evaluating model performance using ROC curves (and their corresponding AUCs) did not provide a clear top-performing model (AUC >0.85) (Figures S4 and S5),^43^ exemplifying the importance of selecting the proper metric for model evaluation. This is particularly striking when considering final model evaluations, where the most permissive ineffective siRNA thresholds showed some of the greatest AUCs (Figure S5, bottom left curves) despite being underfit, as determined by their contingency tables.

### The RF model outperforms the linear classification model built from the same dataset

**Figure 6:**
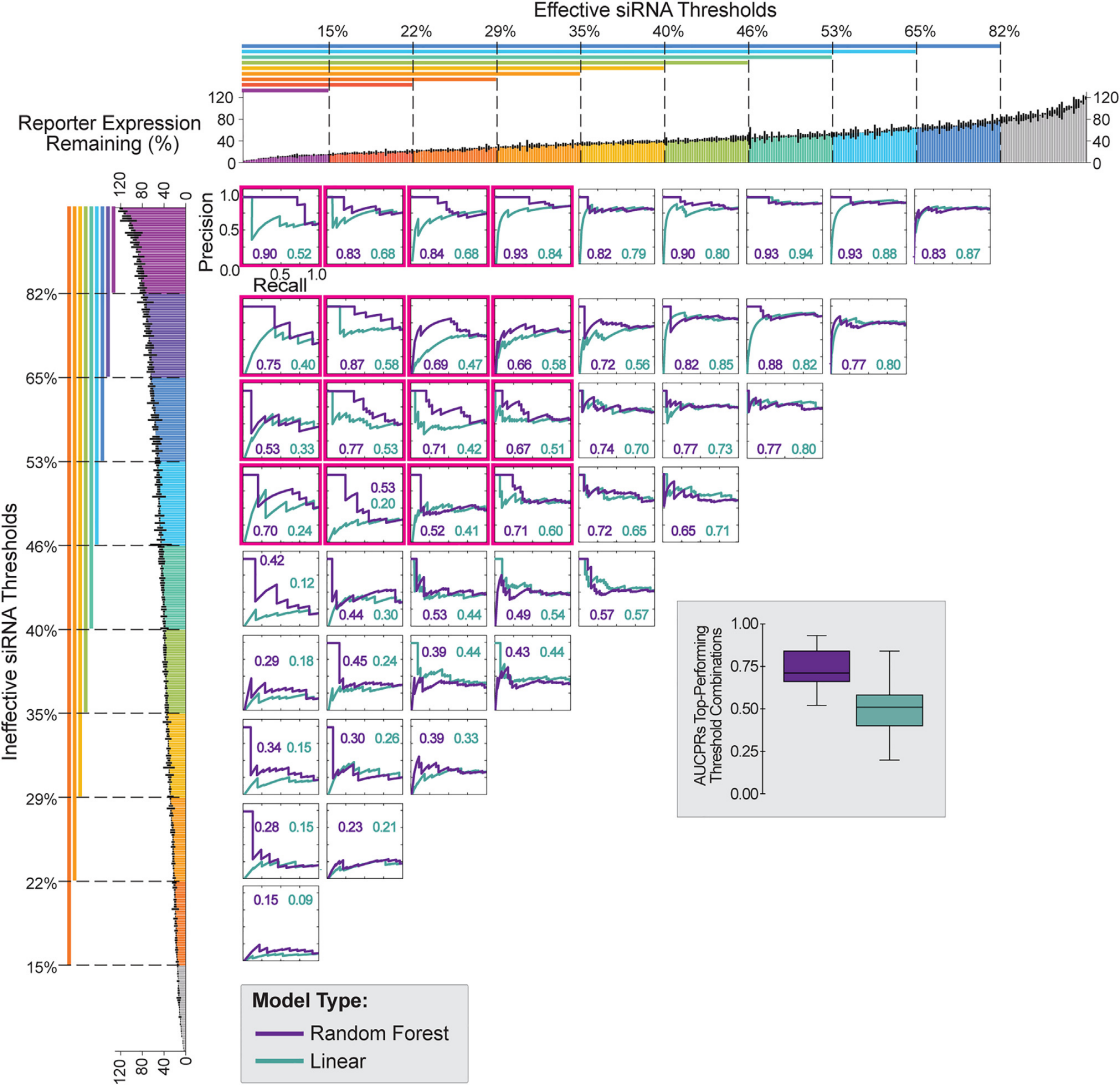
Comparing RF and linear models Precision-recall curves for RF classifiers (purple curves) and linear classifiers (teal curves) trained on the entire training set and evaluated on the holdout set. Each pair of overlaid curves represent performances of models trained using a different E and I siRNA threshold pair. The bar plots at the top and left depict all siRNA target expression data (as in Figure 2D), colored by E (top) or I (left) thresholds. Precision-recall curves are aligned to these bar plots to indicate the E and I thresholds used for training of the corresponding curve’s model. Thresholds are inclusive of all data with expression values less than (for E thresholds) or greater than (for I thresholds) the threshold expression percentage. One can use the AUCPR to evaluate model performance; AUCPRs are indicated in the bottom right corner of each plot and color-coded by model type. The boxplots on the right depict the distribution of AUCPRs for models built with the most stringent thresholds (boxed in pink).

We next compared the performance of RF with a linear model to classify siRNA efficacy in this dataset (Figure 6). For this comparison, we selected a published linear classification method that leverages an *ad hoc* function and utilizes the threshold pairs.^10^ We selected this linear method because it was applied previously to the siRNA dataset used here.^10^ As with the RF model, there was a general trend in higher performance of linear models built with the most stringent threshold pairs (Figure 6, top left curves boxed in pink). Overall, the RF model performed better than the linear model, as determined by higher mean and median AUCPRs for top-performing models (Figure 6, boxplot). While the overall performance of the linear model was significantly worse than that of the RF model, it showed some predictive power with the same top performing thresholds, indicating that elimination of moderate-efficacy siRNAs from model development is beneficial for simple linear models and more sophisticated ML models.

### Visualization of siRNA position base weights driving models by proxy feature extraction

To better understand how one of the top-performing RF models identified effective and ineffective siRNAs, we looked at 20-nt target site position base weights. While deriving this type of matrix is trivial for a linear model, feature extraction from any ML model is complex and model dependent.^47^ Further, most existing methods produce weight magnitudes but not directions (positivity/negativity); thus, while a position base’s contribution to the classification task may be determined, whether it is favored in effective siRNA or disfavored cannot be ruled out.^26^^,^^27^^,^^28^^,^^47^ Existing methods that do provide such information often cannot be applied to all model types or require different approaches depending on the model type, complicating the application.^27^^,^^28^ A frequently applied method introduced by Lundberg et al.^28^ is critically limited computationally, requiring a large amount of computing power that increases exponentially with feature number. This limits evaluation of models built from sequence data, which often contain a large number of features to represent each position base. To overcome these challenges, we devised a method to assign base weights for classifying siRNAs by efficacy that provides a proxy for relative importance and favorability of different feature contributions in decision trees, which is quick to compute and can be applied to any classification model type.

To start, feature vectors for each siRNA used in model development were obtained (materials and methods). Next, the model was used to predict the efficacy of each siRNA. From the known siRNA efficacy and selected h_1_ threshold, each siRNA prediction is placed into one of four classification groups (true positive, true negative, false positive, or false negative). Each of the four classification groups is then considered individually, and the feature vectors of the siRNAs in each group are averaged. The averaged weights of the vectors representing the false positives and false negatives groups are multiplied by −1 because these indicate incorrect predictions. The resulting vectors from the four classification groups are then summed, and the summed vector is transposed back to represent base frequencies at each position in the 20-nt target region (Figure S6).

**Figure 7:**
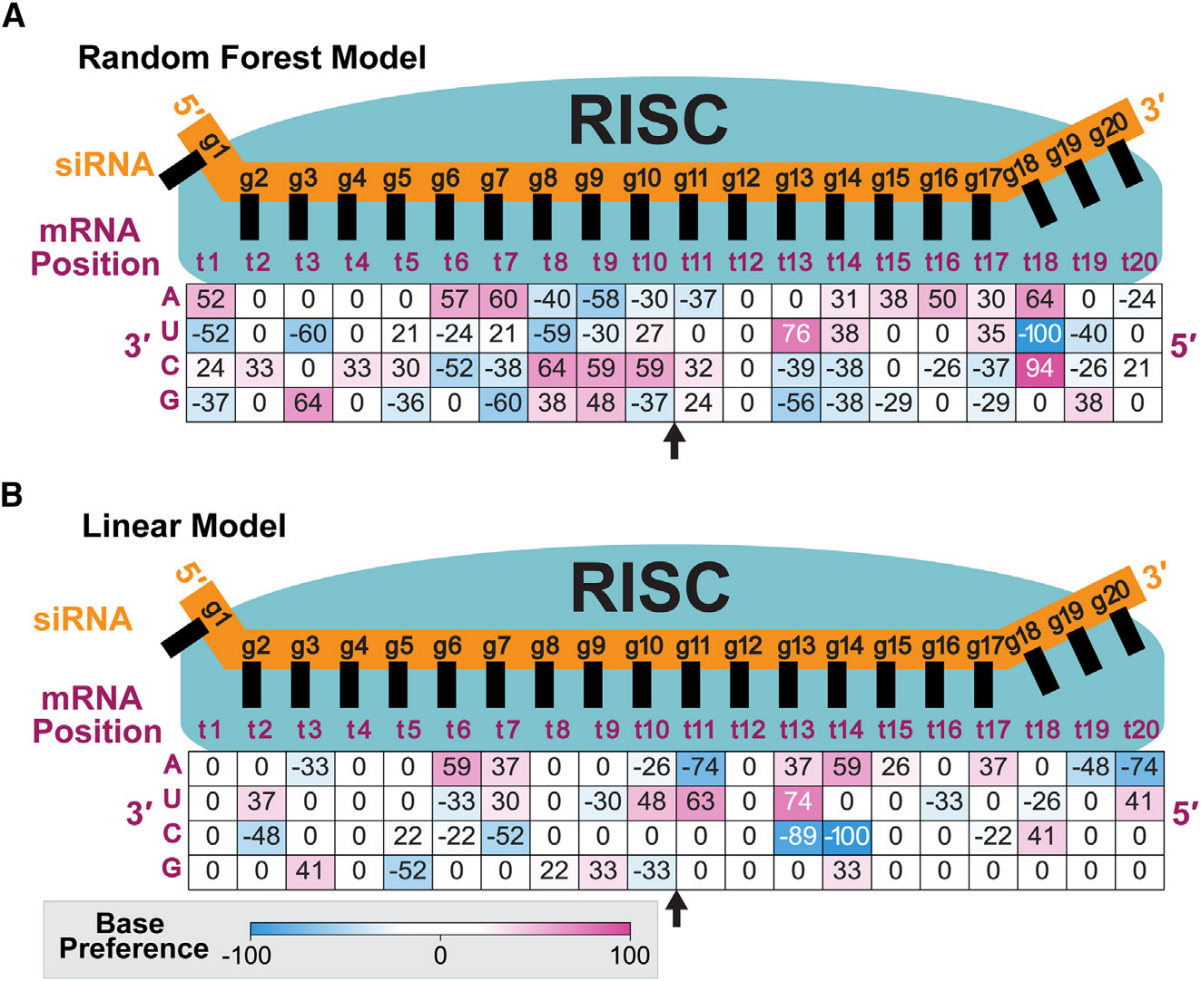
Target site base feature weights identified by siRNA efficacy prediction models (A) Base feature weights extracted from the RF model. Weights were extracted from the 20-nt target site sequence and are aligned with respect to the RISC (Figure 1A) in a matrix by nucleobase indicated in magenta along the left. Positions are indicated for target (t) and guide (g) sequences. Weights are colored by value following the scale indicated. Magnitude indicates importance for the particular model, with higher-magnitude weights indicating bases more important for prediction. Bases with zero weights are not important for prediction for the particular model. Direction (positive/negative) indicates favorability of a base with respect to identifying E siRNAs for the particular model, with positive weights indicating that a feature is favored in identifying E siRNAs and negative weights indicating disfavoring. The model was developed using 22% E and 53% I thresholds. The arrow indicates the mRNA cleavage site between positions t10 and t11. (B) Same as (A) but for a linear model. Base weights were extracted by proxy (results; materials and methods).

The resulting base weights were plotted as a matrix (Figure 7). A base’s importance for classification at a given position with respect to siRNA efficacy prediction is indicated by the weight’s magnitude; a larger-magnitude weight (regardless of whether positive or negative) indicates greater importance, while a weight of zero indicates no importance of that base at that position. For example, in Figure 7A, at position t18, bases A, U, and C with weights of 64, −100, and 94 respectively, all have high importance (with U being the most important), while base G with a weight of 0 is not important. A unique improvement of our method for extraction over other methods is its ability to identify whether a feature is favored or unfavored. Other methods for RF feature extraction only identify a feature’s importance.^47^ A base’s favorability at a given position is indicated by the weight’s direction (positivity or negativity). A positive weight indicates that a base at a particular position is favored in distinguishing effective siRNAs. A negative weight indicates that a base at a particular position is disfavored in distinguishing effective siRNAs. Bases at positions with zero weights have no importance and, thus, are neither favored nor disfavored. For example, in Figure 7A at position t18, bases A, C, and U have high importance, with A and C being favored while U is disfavored. With a weight of 0, base G at position t18 is not important and, thus, neither favored nor disfavored.

This method relies on the output of the classification model and is agnostic to the model type. Therefore, it can be applied to any classification model to extract feature weights. We compared the base weight matrix generated from a linear model by proxy and by direct extraction methods. The two matrices show similar weights, suggesting that the proxy method provides a reasonable approximation of features driving the model performance (Figure S7). Critically, the weights put out by this method are a reflection only of the model from which they were extracted. Direct translation to biological function is tenuous, although the resulting weights can provide insight for developing hypotheses for further experimental evaluation.

### Comparing base weights identified by RF vs. linear classification models

**Figure 8:**
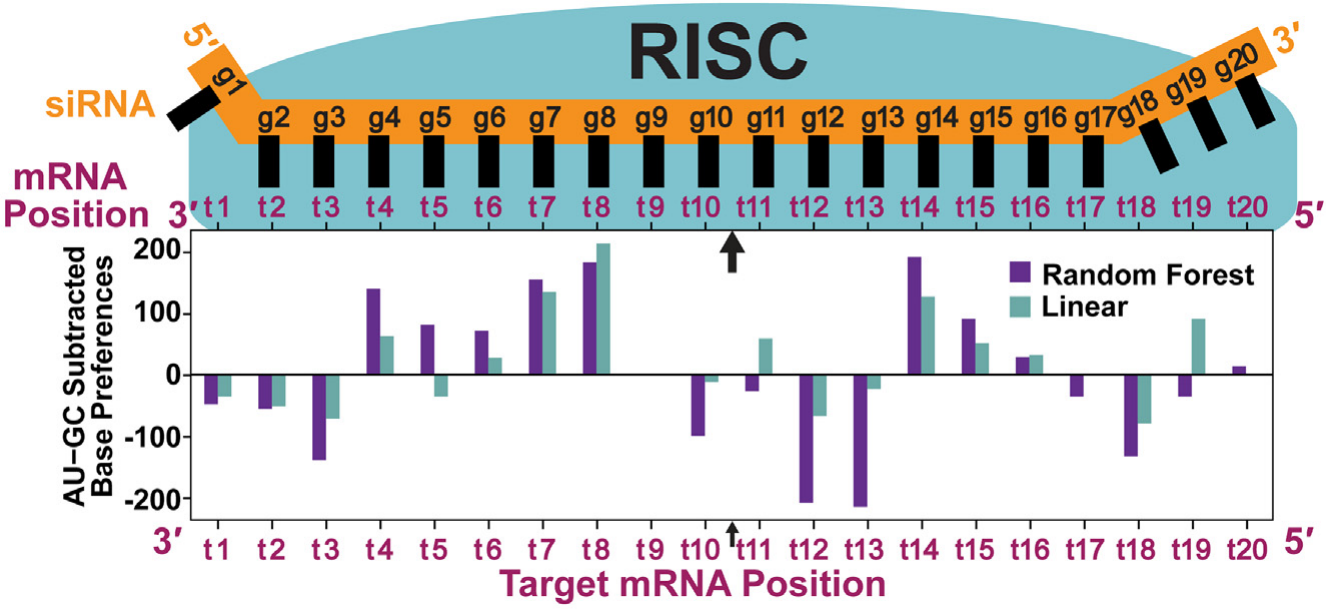
Thermodynamic trends in base weights extracted from the RF and linear models Shown is a comparison of thermodynamic trends approximated by subtracting summed GC weights from summed AU weights extracted from the RF (purple) and linear (teal) models. Weights were extracted from the 20-nt target site sequence and are aligned with respect to the RISC (Figure 1A). Positions in sequences indicated for mRNA target and siRNA guide strands. Base weights were extracted from their respective models using a proxy method (results; materials and methods). Base weight positions are indicated with respect to the position in 20-nt target mRNA sequence (x axis). The arrow indicates the mRNA cleavage site between positions t10 and t11. The linear and RF models from which weights were derived were developed using an E threshold of 22% and an I threshold of 53%. See also Figure S6.

We then analyzed the extracted base weight matrix from linear and RF models built using the same top-performing threshold pair (Figure 7). Overall trends in base weights were similar, with similar AU/GC weights and identical AU/GC favorability (directionality) at 14 positions (Figure 8). RF models produced greater resolution (i.e., the differences between maximal and minimal base importances are greater, enabling greater discrimination), likely explaining better model performance. For both models, there was a trend of no base importance near the seed (guide and target strand mRNA positions 2–5, hereafter written as g2–g5/t2–t5), followed by a region of flexibility (g6–g7/t6–t7), then high affinity near the cleavage site (g8–g11/t8–t11) and high flexibility in the tail (g13–g17/t13–t17). Weaker base importance in the 3′ region (positions t2–t5), which corresponds to the 5′ end of the siRNA seed, likely reflects the need for sequence variability to accommodate a wide range of siRNAs because this region determines siRNA specificity. The lack of specificity here also highlights the flexibility of the model to accommodate a large range of mRNA targets. There is no importance of any base at position t11. Base-pairing in this central region is known to be important for effective cleavage;^48^^,^^49^^,^^50^ therefore, it is possible that the lack of importance at this position is necessary to accommodate different bases in different siRNAs and targets. Even a linear model developed using data from unmodified siRNAs showed low importance of any base at position t11,^10^ suggesting the significance of this position. Absent from these extracted weights is thermodynamic asymmetry, which is critical for nonmodified siRNAs^14^^,^^17^^,^^35^^,^^36^ but is encapsulated in the chemical modification scaffold for the modified siRNAs used in this model.

Thermodynamic trends are intrinsically linked to base weights.^14^ To examine thermodynamic trends, the summed GC weights were subtracted from the summed AU weights at each position (Figure 8).^51^ At all but three positions (t2, t10, and t16) the AU − GC subtracted weight directionalities had the same favorability in both models. In positions of identical directionality, RF frequently had a larger magnitude and, thus, higher importance. Although thermodynamic asymmetry is required for siRNA efficacy,^51^ asymmetry was introduced through chemical modification and structure in this siRNA dataset; thus, does not appear in the weight matrix as a major determinate of efficacy. Overall, base weights (and their corresponding favorabilities and importances) from both models are consistent with the current understanding of siRNA-RISC targeting recognition and cleavage for modified and nonmodified siRNAs.^50^^,^^52^^,^^53^^,^^54^^,^^55^^,^^56^

### Evaluation of models on a randomized siRNA dataset

To further assess the accuracy of our model, we evaluated its performance on randomly designed siRNAs by shuffling the siRNA efficacies with respect to their sequences in the holdout set. The resulting model performance of these randomized siRNAs is poor (Figure S8), indicating that model fitting is, in fact, occurring to relevant sequence information rather than random noise inherently present in such a small dataset.

### Evaluation of the model-building pipeline on external datasets

To determine whether our pipeline can indeed be applied more widely, we applied it on two external sets of nonmodified siRNAs. The first dataset (hereafter referred to as set 1) was evaluated previously by Reynolds et al.^14^ and consisted of 240 siRNA sequences and their corresponding target gene expressions remaining (as a percentage of a control) evaluated in HEK293 cells by either branched DNA assay or luciferase reporter assay (Figure S9A). The second dataset (hereafter referred to as set 2) was evaluated previously by Huesken et al.^57^ and consisted of 2,431 siRNA sequences and their corresponding normalized inhibitory activities evaluated in HeLa cells using a hypoxia response element-luciferase reporter assay (Figure S9B).

Set 2 consisted of approximately 7-fold more (2,431 nonmodified siRNAs) than the data on which the pipeline was developed (356 modified siRNAs). Evaluating our pipeline on such a large dataset may unfairly overestimate our pipeline’s potential. Thus, in addition to applying the pipeline to the full set 2 dataset, we also applied the pipeline to a downsized dataset of 350 randomly selected (materials and methods) sequences (hereafter referred to as set 2 downsized; Figure S9C).

The trichotomous grouping method and pipeline for classification model development (Figure 3) were applied to all three datasets (set 1, set 2, and set 2 downsized), following the identical protocol and parameters applied for modified siRNAs (materials and methods) to produce RF classification models. Effective/ineffective thresholds were selected independently for each dataset by evenly distributing the data by efficacy into 10 groups. The equally distributed efficacy thresholds for set 2 downsized differed slightly from those of the full-size set 2 because of small changes in the distribution of the randomly selected downsized dataset (Figure S9). Model performance was assessed at different threshold combinations by plotting precision-recall curves (Figures S10–S12).

Set 2 was the largest and, thus, as expected, models built from it showed the strongest performance when evaluated on the holdout set (Figure S11). On the two smaller datasets, strong model performance on the holdout sets was seen for several threshold pairs, including 10/49 and 10/61 effective/ineffective for set 1 (Figure S10) and 15/42 and 7/42 for set 2 downsized (Figure S12).

Compared with the dataset on which the model building pipeline was developed, set 2 had a greater representation of effective siRNAs, causing threshold distributions to skew lower (compare Figure 2A with Figure S9B and the bar plots in Figure 5 with Figure S11). Despite this difference, predictive models were achieved, exemplifying the applicability of this framework to datasets that are significantly different in content and distribution. Even within set 2 downsized, this shifted distribution of efficacies remained, and predictive models were able to be developed using the same pipeline (Figure S9).

All three external datasets produced a pattern of model performance similar to that seen with models built with modified siRNAs (Figure 5), in which models built with the second to third lowest effective thresholds first and third highest ineffective thresholds produced models with strong performance. This indicates robustness in the thresholding method because thresholds were selected independently for each dataset by evenly distributing the data by efficacy into 10 groups. This demonstrates the applicability of the trichotomous data partitioning method and model-building pipeline presented in this manuscript to other datasets of a similar scale to enable development of predictive ML models from limited datasets.

The features for a top-performing model from each of the three external sets (built from the 10/61, 14/49, and 15/42 effective/ineffective threshold pairs for the set 1, set 2, and randomly set 2 downsized datasets, respectively) were extracted (materials and methods), and the resulting base weights were visualized (Figures S13–S15). The structure of the nonmodified siRNAs from sets 1 and 2 differed from the modified siRNAs, consisting of a 21-nt duplex with two deoxynucleotide overhangs on the 3′ terminus.^14^^,^^57^ The resulting difference in guide strand length is depicted in the RISC schematic in Figure S15. The models were built using the 20-nt target site, enabling direct comparisons of base weights across the models. While base weights from each model show some similarity with those extracted from the model built with modified siRNA (Figure 7), overall trends are quite different. The weights extracted from the set 1 model show little importance in the seed region. Across all three sets there is a moderate trend of AU favoring and GC disfavoring in the 3′ region of the target site and the opposite trend in the 5′ region. Features extracted from Set 2 and its randomly downscaled set 2 downsized show general trends in similarities but also differ at several positions, with the features derived from the larger set 2-produced model having a greater number of positions with high importance.

### Experimental evaluation of the pipeline on a panel of synthesized siRNA

To experimentally evaluate our pipeline in the context of siRNA design, we applied our top-performing RF model (built using 22% effective and 53% ineffective thresholds; Figure 5) to classify compounds targeting transcripts from four human genes: *MAPT*, *APP*, *SNCA*, and *BACE1* (materials and methods).

The 10 siRNAs with the highest confidence scores predicted to be effective and the 10 siRNAs with the lowest confidence scores predicted be ineffective were selected for experimental evaluation (Table S3). siRNAs were synthesized with the 20 selected targeting region sequences, and their silencing efficacies were experimentally evaluated using a dual-luciferase reporter assay system (materials and methods; Figure S16A). Of the 10 compounds predicted to be effective, 7 were truly functional. Of the 10 compounds predicted to be ineffective, 8 were truly nonfunctional (Figure S16B). These results indicate that the RF model developed using the framework presented here can be applied to successfully identify functional siRNAs.

## Discussion

In this paper, we provide a framework for simple application of ML to small biological datasets. This framework uses a noncanonical trichotomous partitioning method that explores a range of classification thresholds to overcome data variability, uncertainty, and noise common to small biological datasets, a K-fold cross-validation method for training ML models to small datasets that can be adapted (e.g., changing partition size or K size) to a range of biological problems, and a novel evaluation metric that accounts for data imbalances and varying classification thresholds to enable simple performance comparisons across models. Finally, we present a novel method to extract feature weights from any classification model by proxy. This framework is presented through the lens of siRNA design but is applicable to any small, variable biological dataset, providing a tool to tap into a previously inaccessible resource to advance biological knowledge.

When using modeling for analysis of complex biological datasets, it is essential to consider the data and the question(s) seeking to be answered in a holistic way. For siRNA design, we selected a classification model type despite siRNA efficacy data being continuous, which is typically better suited for regression. Indeed, many existing siRNA design models apply regression.^17^^,^^36^^,^^58^^,^^59^^,^^60^

However, regression models rely greatly on moderate-efficacy siRNAs, which are not well understood but are likely limited by ineffective RISC loading, target release,^52^^,^^61^^,^^62^ and other complex components that sequence-centered models cannot capture and cannot be determined from efficacy data alone. Thus, fitting a regression model to these data introduces a large amount of uncertainty into the model, reducing its predictive power. Using classification improves existing siRNA prediction models by allowing the model to use data with a clearer underlying mechanism: effective and ineffective siRNAs. In fact, we found that all of the identified top-performing models excluded moderate-efficacy siRNAs from training.

A major challenge of building a classification model with continuous, inherently noisy siRNA efficacy data is distinguishing data points near a classification threshold. Our trichotomous classification system employs two thresholds, one to define effective siRNA and one to define ineffective siRNA, to eliminate noise overlap and enable proper separation of the siRNA classes. By considering a range of effective and ineffective threshold pairs (45 total combinations), we overcome bias associated with selecting a threshold in continuous data not typically amenable to binary classification. We found that the most stringent thresholds, which eliminate up to 80% of the training data, provided models with the greatest performance, exemplifying a tradeoff between accuracy and coverage in model building. With a range of thresholds to choose from, we were able to balance satisfactory performance with inclusion of sufficient data to capture biological features driving siRNA efficacy. Trichotomous partitioning also provides greater power over threshold definition, enabling tuning of a model specifically to the problem at hand. For example, by picking a stringent h_1_ threshold and a less stringent h_2_ threshold, the model will more heavily weigh effective siRNAs, enabling a stronger ability to identify them over ineffective siRNAs, which are mostly irrelevant to the biological problem at hand.

Proper model evaluation is a critical step in model building because it identifies a highly predictive model among weaker models. Here, AUCPR_adj_ adjusts the commonly used AUCPR by the P_R=1_ to provide a single, easy-to-compare numeric metric that enables performance comparisons across imbalanced datasets and multiple h_1_ thresholds, something other existing model evaluation metrics cannot do. The ability to tailor evaluation metrics to the biological data and question being considered enables proper tuning of a model to optimize its performance. For siRNA design, AUCPR_adj_ prevents overestimation of model performance and ensures proper model assessment to guide model tuning and final model selection so that the final model performs well when applied in decision-making.

We successfully developed a supervised ML model that predicts siRNA efficacy with higher power than a simpler linear model (using the same classification threshold pairs), confirming the power of ML models when applied to biological problems. When assessed experimentally, this ML model performed well, with 7 of the 10 siRNAs predicted to be effective showing potent silencing (<22% reporter expression remaining) when evaluated in cells using a dual-luciferase reporter assay. This improvement exemplifies the power of ML in improving prediction compared with simpler models. Critically, this improvement does not indicate that the model presented here is superior to existing siRNA design models; rather, this performance improvement highlights the value of this approach when applied to limited datasets.

However, the complexity of ML models makes it challenging to elucidate how they fit the data. This poses a major challenge in cases where the models fit to irrelevant patterns in the data, leading to development of a poor-quality model that shows high performance.^21^ Treating such models as “black boxes” limits insight into the biological mechanism the model seeks to describe. The feature extraction method presented here is a simple, quick-to-compute method for extracting proxy features from a model to determine feature importances. Feature importances provide valuable insight into a model from which hypotheses for biological mechanism can be developed to be later evaluated experimentally. This unlocks the potential of using (and examining) more complex models to address important biological questions. Moreover, because our method is evaluation focused, relying only on the classification outputs of a model, it is simple to apply and adapt to any model type. Having a universal methodology to quickly examine a range of models from the simplest linear models to the most complex deep learning models will shed light onto the black box of a model’s predictive mechanism.

Feature base weights (and their corresponding base importances) from a selected top-performing ML model reflect the current understanding of the RISC mechanism (applicable to modified and nonmodified siRNAs), such as high affinity at the cleavage site and variability 3′ to the cleavage site (position t11), demonstrating the ability of the model to accurately recapitulate biology. Absent from these preferences was thermodynamic asymmetry, which is critical for proper strand loading into the RISC and is a major component of many existing design algorithms for nonmodified siRNAs.^14^^,^^17^^,^^35^^,^^36^^,^^51^ This is expected because this asymmetry was incorporated through the chemical scaffold and structure of the modified siRNAs used to build the model (Figure 1B). When applying our feature extraction method to models built with external nonmodified siRNA datasets, a moderate trend of AU favoring and GC disfavoring in the 3′ region of the target site is seen, with the opposite trend in the 5′ region (Figures S13–S15). This shows some correlation with the thermodynamic asymmetry requirements important for proper strand loading of nonmodified siRNAs into the RISC.^14^^,^^17^^,^^35^^,^^36^ While valuable for developing hypotheses, extracting feature weights (and corresponding importances and favorabilities) is model specific and may not directly reflect biological function. Feature extraction from a model also has the potential to introduce bias. The method presented here for feature extraction faces potential limitations, particularly in the context of correlated features, which it may fail to discriminate.

Features extracted from the linear and ML models show similarities in feature weights, with ML achieving higher resolution. The linear model is constructed by subtracting base frequencies, and therefore the features will closely reflect the base weights of the model (Figure S17).^10^ While the RF model generally aligns with base weights of the linear model, it also reflects more complex interactions. This is because the linear model considers each base and position entirely independently, whereas the RF model can model feature dependencies through its branching structure.^22^ In many base positions where the models differ, the linear model has a weight of zero. This could reflect the linear model’s simplicity (and therefore weakness) in fitting the complex data, leading to washing out of some base importances (i.e., causing the weights to decrease in magnitude). Moreover, when considering thermodynamic trends derived from feature weights, both models recapitulate significance of flexibility in the tail that is shown to promote product release (Figure 8).^52^^,^^61^ However, RF frequently had larger magnitude trends, which may indicate that the more complex RF model is better at accounting for thermodynamic effects. Our findings suggest the value of applying ML models to smaller biological datasets because the resulting models can be more biologically accurate and informative.^22^ While the model itself, not the feature weight matrix, is used for predicting siRNA efficacy, future analysis of feature dependencies and potentially including multiple position bases in a single feature could further improve the model.

siRNAs are coming of age. Advances in chemical modification have enabled siRNA delivery to many tissues (liver, kidney, and brain).^11^ A critical next step is identifying effective sequence targets for these siRNA chemistries. Accurate methods to streamline the design and validation of chemically modified siRNAs are needed to complete this task. By applying the framework presented here using well-documented and ready-to-use ML packages like Scikit-Learn,^63^ or software tools like Weka that require no coding knowledge,^64^ a wide range of scientists can now harness the power of ML to simplify siRNA drug development.

## Materials and methods

### Dataset acquisition

The siRNA sequences and corresponding efficacy data used in this analysis were obtained from a publicly available dataset.^10^ These siRNAs were evaluated for their efficacy in target gene silencing in HeLa cells using a dual-luciferase assay as described previously.^10^

### Efficacy threshold selection

Pairs of thresholds (effective [h_1_] and ineffective [h_2_]) were selected so that they were evenly distributed from the lowest reporter expression value in the dataset (4%) to the highest (120%) to maintain approximately the same number of points within each group (between 23 and 24). Nine h_1_ thresholds were defined to include siRNAs with report expression values equal to or less than 15%, 22%, 29%, 35%, 40%, 46%, 53%, 65%, and 82%. The nine h_2_ thresholds were defined with the same distribution and included all siRNAs with reporter expression values greater than 15%, 22%, 29%, 35%, 40%, 46%, 53%, 65%, and 82%.

In the trichotomous partitioning scheme presented in this manuscript, siRNAs with reporter expression values greater than or equal to the effective threshold but less than or equal to the ineffective threshold were classified as undefined. All permutations of non-overlapping threshold pairs were considered (i.e., h_1_ ≤ 15%, h_2_ >15% was considered but h_1_ ≤ 35% and h_2_ >22% was not).

### Feature parameterization

For each siRNA sequence, the 20-nt target site of the target mRNA was extracted (Figure 1A) to generate feature vectors for training the model. Specifically, the sequences were encoded into basic binary features using the following protocol. As were represented as [1,0,0,0], Us were represented as [0,1,0,0], Cs were represented as [0,0,1,0], and Gs were represented as [0,0,0,1]. For each sequence, arrays of encoded bases were appended in the order in which they appear in the sequence to form the final 80-dimensional feature vector, representing the full 20-nt target site sequence. Feature vectors were labeled with previously described activity classifications (effective/ineffective/undefined).

### Assessment protocol

A training set containing 75% of the data (267 siRNAs) and a holdout dataset containing 25% (89 siRNAs) were randomly selected using the Scikit-Learn train_test_split method, which ensured unbiased random partitioning of data into desired proportions and enabled equal distribution of effective and ineffective siRNAs when changing the h_1_ and h_2_ thresholds.^63^

The training dataset was used in K-fold cross-validation, during which it was partitioned randomly into 10 K groups using the Scikit-Learn KFold method, which ensured random and even partitioning of the data.^63^ To ensure that all 267 siRNAs from the training set were included in cross-validation, six groups contained 30 siRNAs, and three groups contained 29 siRNAs. During partitioning of K groups, dataset classification was considered to ensure that test groups were balanced with approximately equal numbers of effective and ineffective siRNAs.

Model performance was assessed by AUCPR_adj_ measure. To compute AUCPR_adj_, the precision-recall curves were plotted using the Matplotlib package,^65^ followed by computing the AUCPR values using the auc function from Scikit-Learn.^63^ Values of AUCPR_adj_ were normalized to reflect the range from 0–100. The color scheme was designed to follow the same range from 0 (blue) to 100 (yellow).

### ML model training

Using the feature vectors, the supervised learning models were trained using the RF classifier from the Scikit-Learn Python package.^63^ All RF models were trained with the following default parameters: 200 total trees with a maximum tree depth of 3 nodes and at least one sample per leaf. Model performance was also evaluated solely on the training set during training (Figures S17 and S18). The linear classifier models were trained using a published method that leverages an *ad hoc* function and the three activity classification groups.^10^ This linear method was selected because it was previously applied to the siRNA dataset used here.^10^

### Position base weight determination

The direct feature extraction method applied previously to a linear model is not applicable in the case of more advanced non-linear RF model;^10^ therefore, we developed an alternative “proxy” feature extraction method that is agnostic to the model type (results). When applied to the linear model, the new feature extraction method shows comparable performance determining the same significant features (Figure S7). Feature weights from the linear and RF models, regardless of derivation method, were normalized between −100 and 100, maintaining 0 as the middle weight indicating no importance. Feature weights between −20 and 20 for all models were set to 0 to minimize noise from low-weight features.

### Data preparation for model evaluation on external datasets

The 20-nt target site sequences from the siRNAs evaluated previously by Reynolds et al.^14^ (set 1) were extracted from the published 19-nt antisense strand sequences by determining the reverse complement of the first 20 nt of the corresponding target transcript sequence. The 20-nt target site sequences from the siRNAs evaluated previously by Huesken et al.^57^ (set 2) were extracted from the published 21-nt guide strand sequences by determining the reverse complement of the first 20 nt.

Set 2 siRNA efficacies were converted from normalized inhibitory activities to percentages by dividing all values by the maximum normalized inhibitory activity. Set 2 downsized was generated from set 2 using the Python random.choice method to randomly select 350 siRNAs. For the downsized dataset, the normalized inhibitory activities were converted to percentages by dividing all values by the maximum normalized inhibitory activity within the randomly selected set.

### Applying the ML model for selecting siRNAs for experimental evaluation

Transcript sequences from four human genes (*APP*, *MAPT*, *BACE1*, and *SNCA*; NCBI: NM_000484, NM_001377265, NM_012104, and NM_000345) were selected as target transcripts for selection of siRNA target sequences. The transcript sequences were split into 20-mers using a sliding window of 20 nt to produce targeting region sequences, which were then one-hot encoded as described above. The RF model generated using the 22/53 threshold pair was applied to the encoded sequences using the Scikit-Learn predict_proba method to obtain the binary prediction (effective or ineffective) as well as a confidence score for each targeting region.^63^ The targeting region sequences were grouped into predicted effective and predicted ineffective groups, and each group was sorted by confidence score. The top 10 sequences from each group were selected for experimental evaluation (Table S3). Modified (scaffold depicted in Figure S19) siRNAs were synthesized to target these the corresponding 20-nt targeting region sequences. The siRNA antisense strands were designed so that they were complementary to their corresponding target sequence, with the 5′ U being held constant.

### Experimental efficacy evaluation of model-selected siRNAs

A dual-reporter assay was performed in HeLa cells to evaluate siRNA efficacy as described previously.^10^ Reporter plasmids for each target gene were constructed with fusions of the 20-nt targeting region sequences inserted into a psiCHECK-2 vector (Promega, C8021).

## Data Availability

The data supporting the findings of this study are available within the manuscript and Supplemental Material file.

## Supplemental information

Document S1. Figures S1–S19 and Tables S1–S3

Document S2. Article plus supplemental information
