# Supplementary material for: Asymmetric trichotomous partitioning overcomes dataset limitations in building machine learning models for predicting siRNA efficacy
Source: Mol Ther Nucleic Acids. 2023 Jun 14;33:93–109. doi: 10.1016/j.omtn.2023.06.010 (PMC10338369; doi:10.1016/j.omtn.2023.06.010)
Supplement: Document S1. Figures S1–S19 and Tables S1–S3 [file mmc1.pdf]

OMTN, Volume *33*

Supplemental information

Asymmetric trichotomous partitioning overcomes dataset limitations in building machine learning models for predicting siRNA efficacy

Kathryn R. Monopoli, Dmitry Korkin, and Anastasia Khvorova

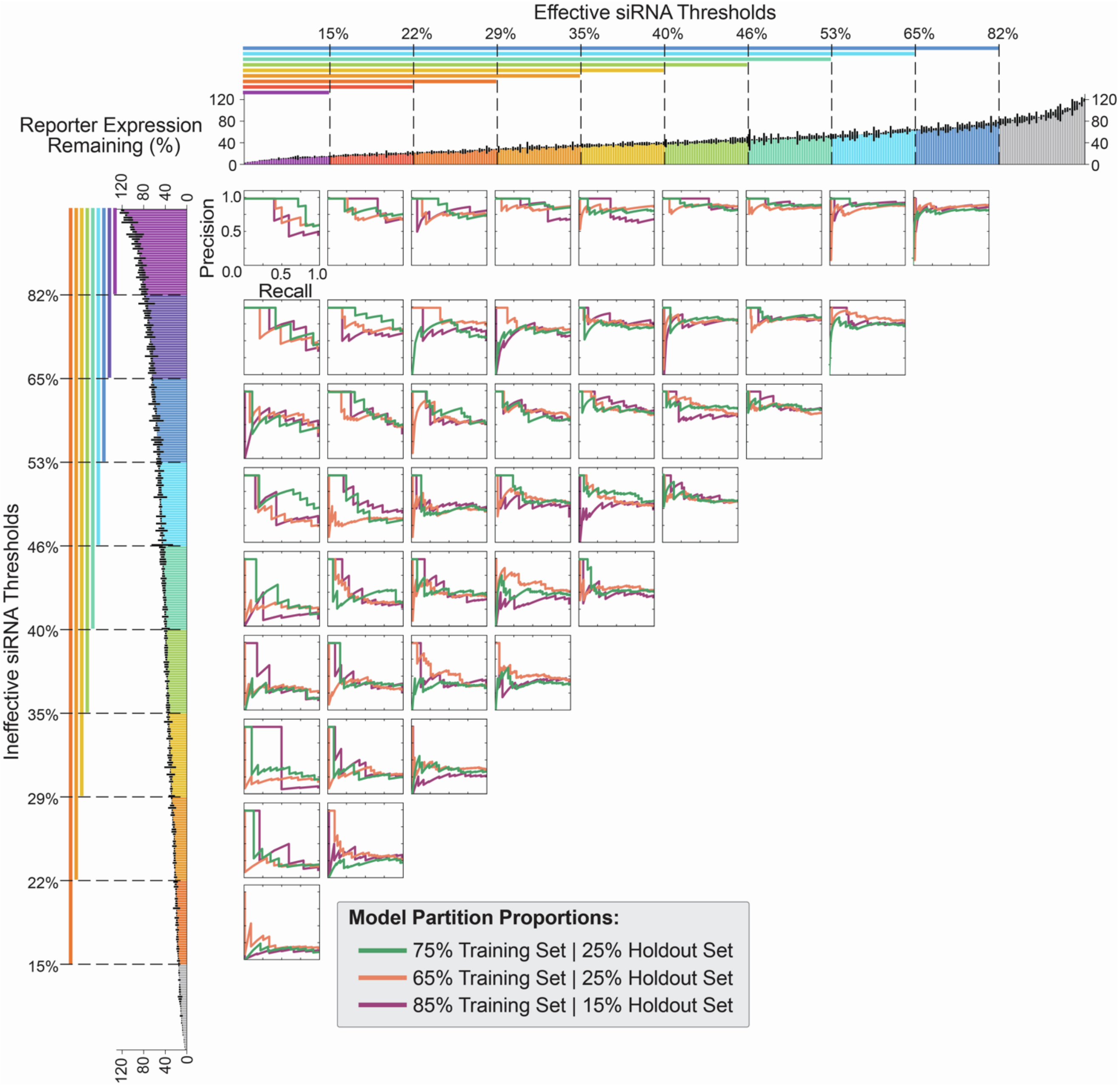

Figure S1. Model performance per classification threshold comparing different training and holdout partition proportions. Trichotomous partitioning and supervised machine learning model building framework (outlined in Figure 3, see Results and Methods) was applied with different training/holdout set proportions: 75%/25% (green), 65%/35% (orange), 85%/15% (purple). Precision-recall curves for model performance during evaluation on the respective holdout sets are shown. Random forest classifiers were trained on entire training set and evaluated on holdout set. Each plot represents performance of models trained using different effective and ineffective siRNA threshold pairs. Bar plots at top and left depict all siRNA target expression data (as in Figure 2D) colored by effective (top) or ineffective (left) thresholds. Precision-recall curves are aligned to these bar plots to indicate the effective and ineffective thresholds used for training of the corresponding curve's model. Thresholds are inclusive of all data with expression values less than (for effective thresholds) or greater than (for ineffective thresholds) the threshold expression percentage.

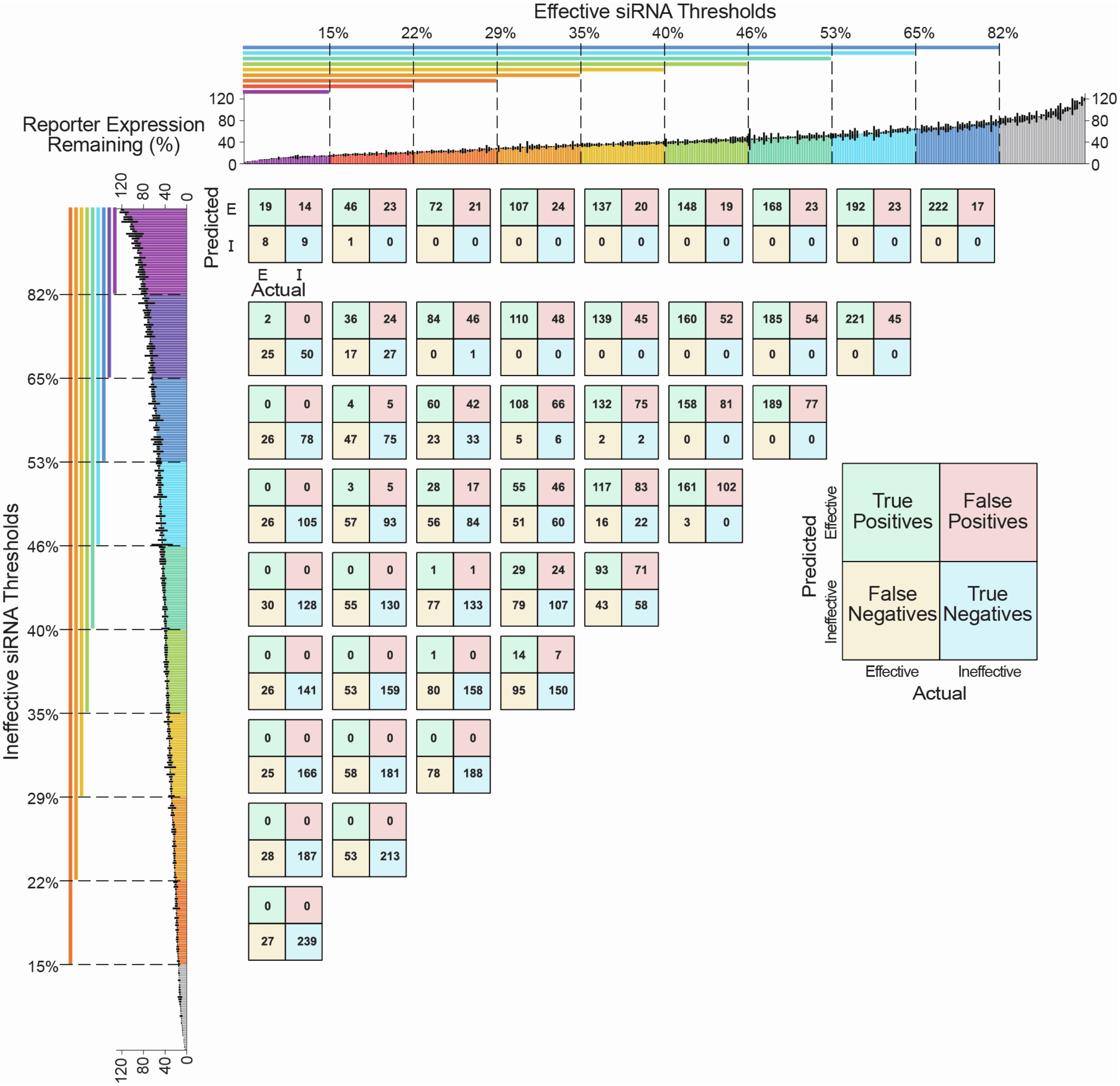

Figure S2. Contingency tables from K-fold cross-validation per classification threshold, related to Figures 5 and S4. Contingency tables depicting the distribution of prediction classes of actual and random forest classifier-predicted siRNA efficacies. Each table represents a single random forest classifier trained with different effective and ineffective siRNA threshold combinations evaluated on the Kth test set. Evaluations on each Kth subset were averaged over all K (K=10) rounds of cross-validation. Tables are color-coded to depict classification group type as indicated in the example larger table on the right. Bar plots at top and left depict all siRNA target expression data (as in Figure 2D) colored by effective (top) or ineffective (left) thresholds. Tables are aligned to these bar plots to indicate the effective and ineffective thresholds used for training of that curve's classifier. Thresholds are inclusive of all data with expression values less than (for effective thresholds) or greater than (for ineffective thresholds) the threshold expression percentage. Grey bars indicate siRNAs excluded from model training for the indicated classification (effective or ineffective). Contingency tables were built at the 0.5 confidence margin for all models.

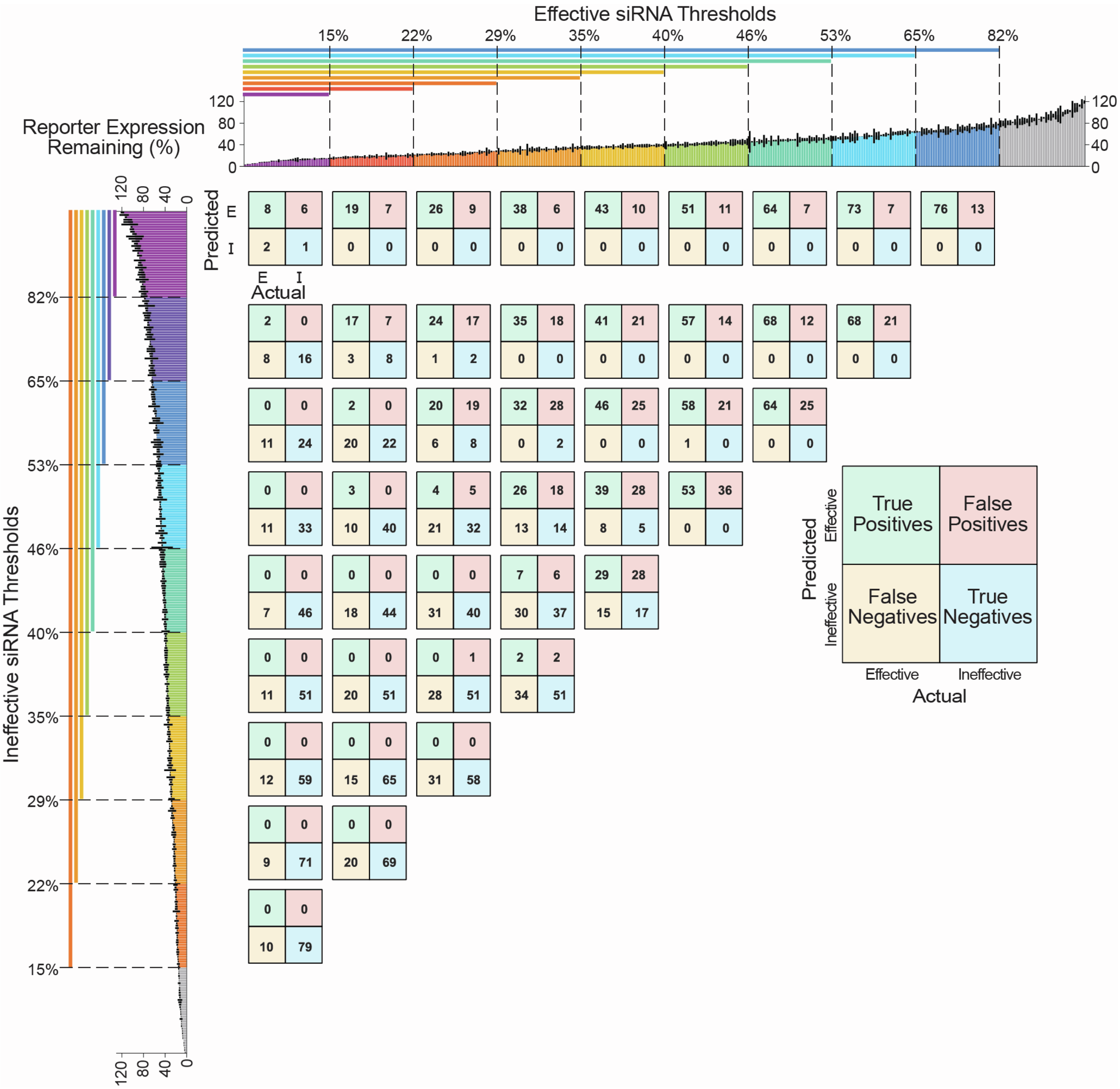

Figure S3. Contingency tables per classification threshold, related to Figures 5 and S5. Contingency tables depicting the distribution of prediction classes of actual and random forest classifier-predicted siRNA efficacies. Each table represents a single random forest classifier trained with different effective and ineffective siRNA threshold combinations evaluated on the holdout dataset. Tables are color-coded to depict classification group type as indicated in the example larger table on the right. Bar plots at top and left depict all siRNA target expression data (as in Figure 2D) colored by effective (top) or ineffective (left) thresholds. Tables are aligned to these bar plots to indicate the effective and ineffective thresholds used for training of that curve's classifier. Thresholds are inclusive of all data with expression values less than (for effective thresholds) or greater than (for ineffective thresholds) the threshold expression percentage. Grey bars indicate siRNAs excluded from model training for the indicated classification (effective or ineffective). Contingency tables were built at the 0.5 confidence margin for all models.

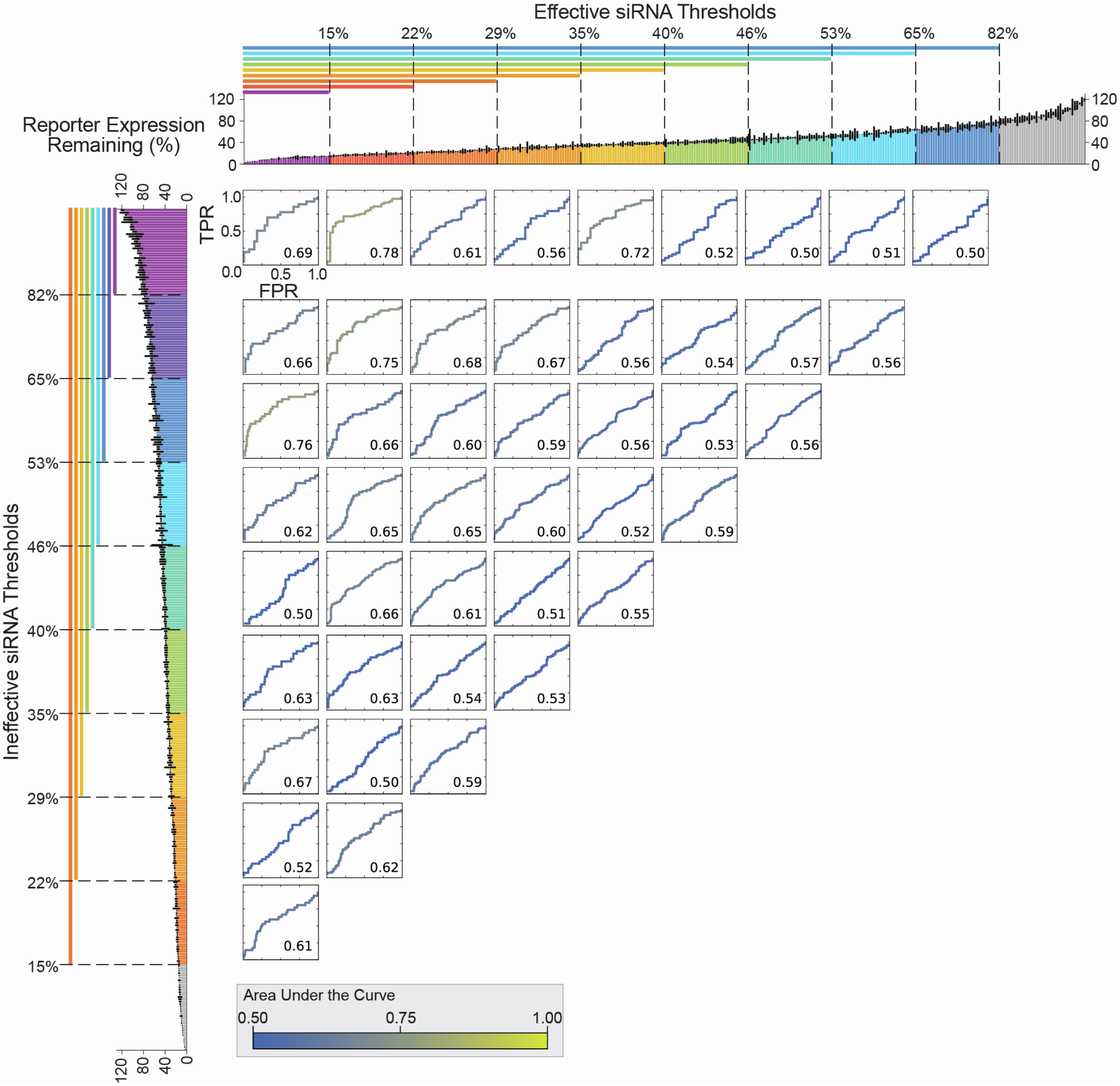

Figure S4. Receiver operating characteristic curves from K-fold cross-validation, related to Figures 5 and S2. True positive rate (TPR) plotted against false positive rate (FPR) of random forest classifiers evaluated on the Kth test set. Evaluations on each Kth subset were averaged over all K (K=10) rounds of cross-validation. Each curve represents a single random forest classifier trained with different effective and ineffective siRNA threshold combinations. Curves are colored by the area under the curve. Color bar depicts area under the curve. Bar plots at top and left depict all siRNA target expression data (as in Figure 2D) colored by effective (top) or ineffective (left) thresholds. Curves are aligned to these bar plots to indicate the effective and ineffective thresholds used for training of that curve's classifier. Thresholds are inclusive of all data with expression values less than (for effective thresholds) or greater than (for ineffective thresholds) the threshold expression percentage. Grey bars indicate siRNAs excluded from model training for the indicated classification (effective or ineffective). Area under the curve indicated in bottom right corner of each plot.

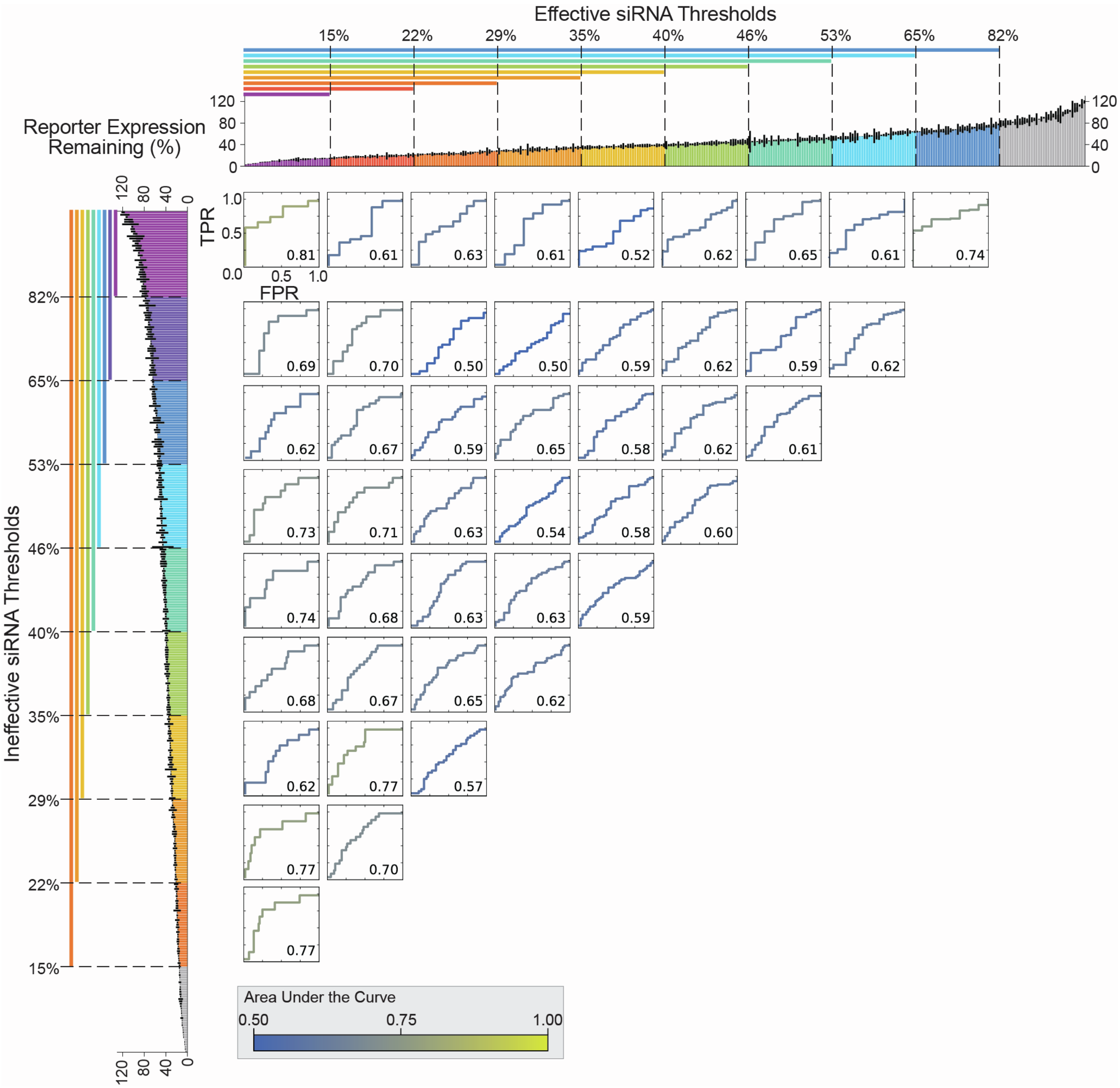

Figure S5. Receiver operating characteristic curves per classification threshold, related to Figures 5 and S3. True positive rate (TPR) plotted against false positive rate (FPR) of random forest classifiers evaluated on the holdout dataset. Each curve represents a single random forest classifier trained with different effective and ineffective siRNA threshold combinations. Curves are colored by the area under the curve. Color bar depicts area under the curve. Bar plots at top and left depict all siRNA target expression data (as in Figure 2D) colored by effective (top) or ineffective (left) thresholds. Curves are aligned to these bar plots to indicate the effective and ineffective thresholds used for training of that curve's classifier. Thresholds are inclusive of all data with expression values less than (for effective thresholds) or greater than (for ineffective thresholds) the threshold expression percentage. Grey bars indicate siRNAs excluded from model training for the indicated classification (effective or ineffective). Area under the curve indicated in bottom right corner of each plot.

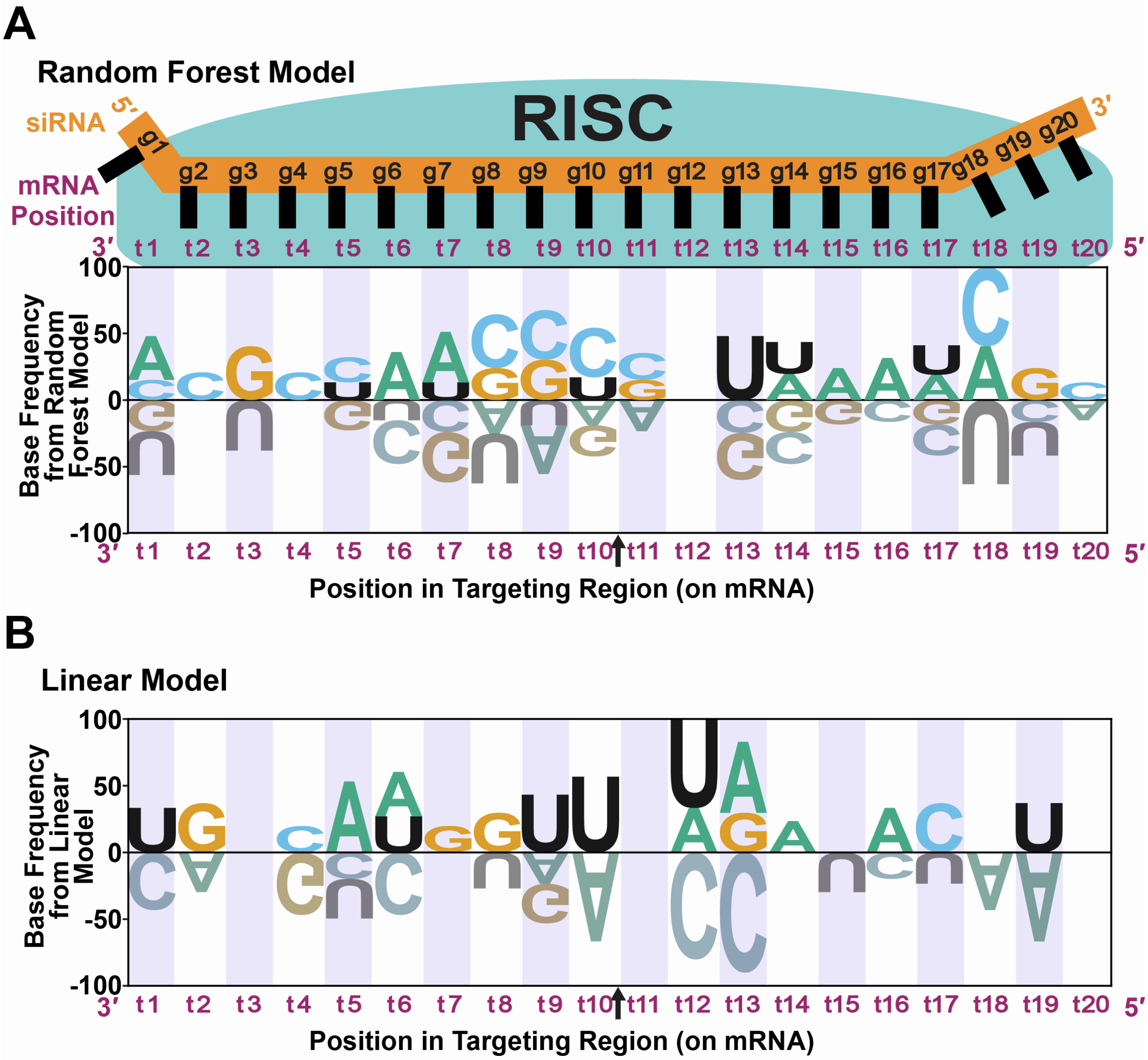

Figure S6. Target site base feature weights identified by siRNA efficacy prediction models depicted as sequence logos, related to Figure 8. Base feature weights extracted from (**A**) random forest machine learning model or (**B**) linear model using proxy base weight extraction method (see Results and Methods). Positions in sequences indicated for mRNA target (t) and siRNA guide (g) strands. Letter heights indicate importance of base weights to siRNA efficacy prediction at each position for the particular model; taller letters indicate bases with greater importance, shorter letters indicate those less important. Letter direction (positive/negative) indicates favorability of a base with respect to identifying effective siRNAs for the particular model, with positive weights indicating a feature is favored in identifying effective siRNAs, and negative weights indicating disfavoring. Both random forest and linear models were developed using 22% effective and 53% ineffective thresholds respectively. Arrow indicates mRNA cleavage site between t10 and t11. Data depicted here are identical to those presented in Figure 8, but in sequence logo form rather than matrix form. Feature extraction performed at the 0.5 confidence margin.

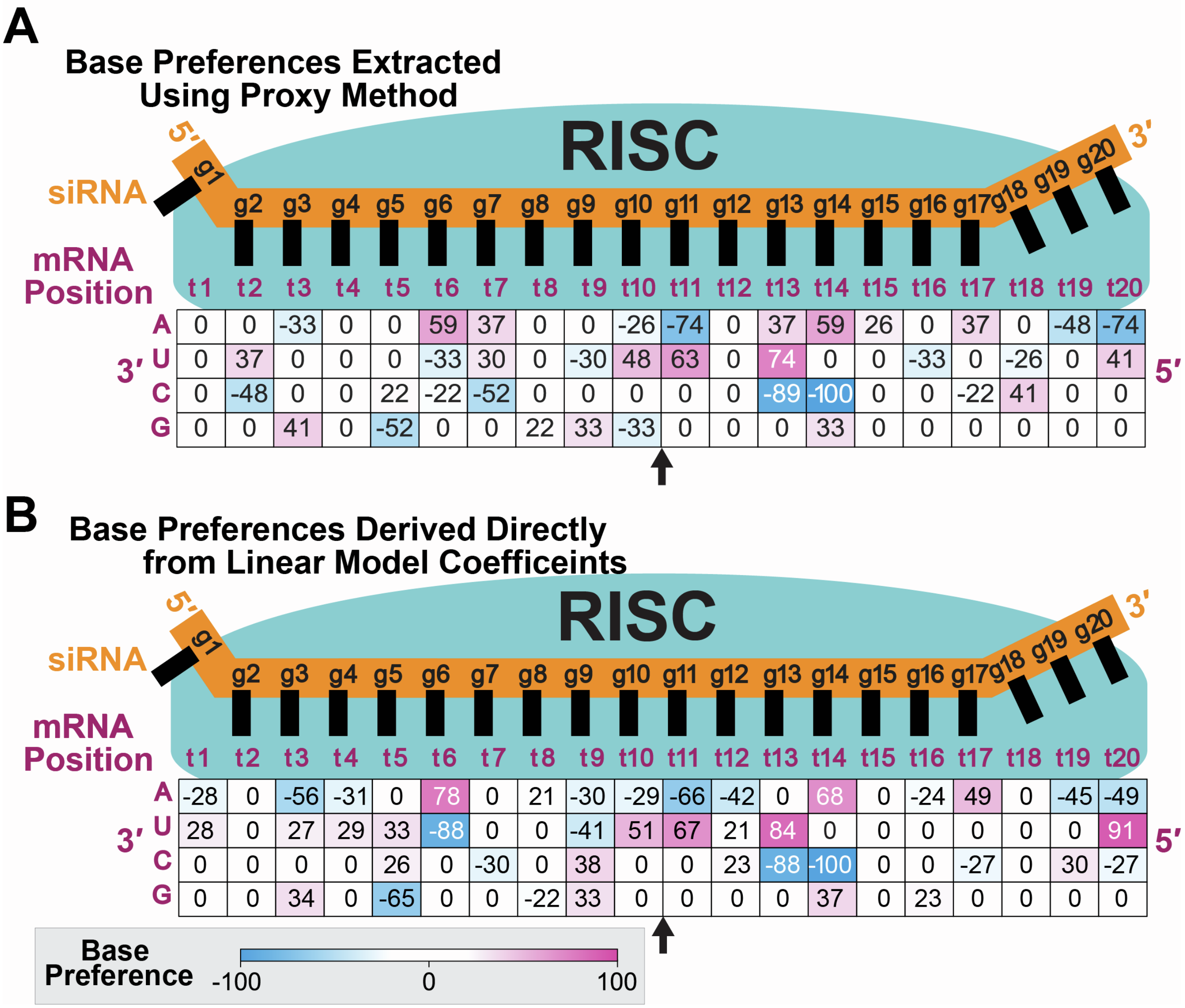

Figure S7. Proxy method for base feature weight extraction shows high correlation with weights directly extracted from linear model. Base feature weights extracted from the same linear model using (**A**) the proxy feature extraction method and (**B**) directly from the linear model coefficients (see Results and Methods). Weights were extracted from the 20 nt target site sequence and are aligned with respect to the RNA-induced silencing complex (RISC) (see Figure 1A) in a matrix by nucleobase indicated in magenta along the left. Positions in sequences indicated for mRNA target (t) and siRNA guide (g) strands. Weights are colored by value following the scale indicated at bottom. Magnitude indicates importance for the particular model, with higher magnitude weights indicating bases more important for prediction. Bases with zero weights are not important to prediction for the particular model. Direction (positive/negative) indicates favorability of a base with respect to identifying effective siRNAs for the particular model, with positive weights indicating a feature is favored in identifying effective siRNAs, and negative weights indicating disfavoring. Both matrices were derived from the same linear model developed using 22% effective and 53% ineffective thresholds respectively. Arrow indicates mRNA cleavage site between t10 and t11. Feature extraction performed at the 0.5 confidence margin.

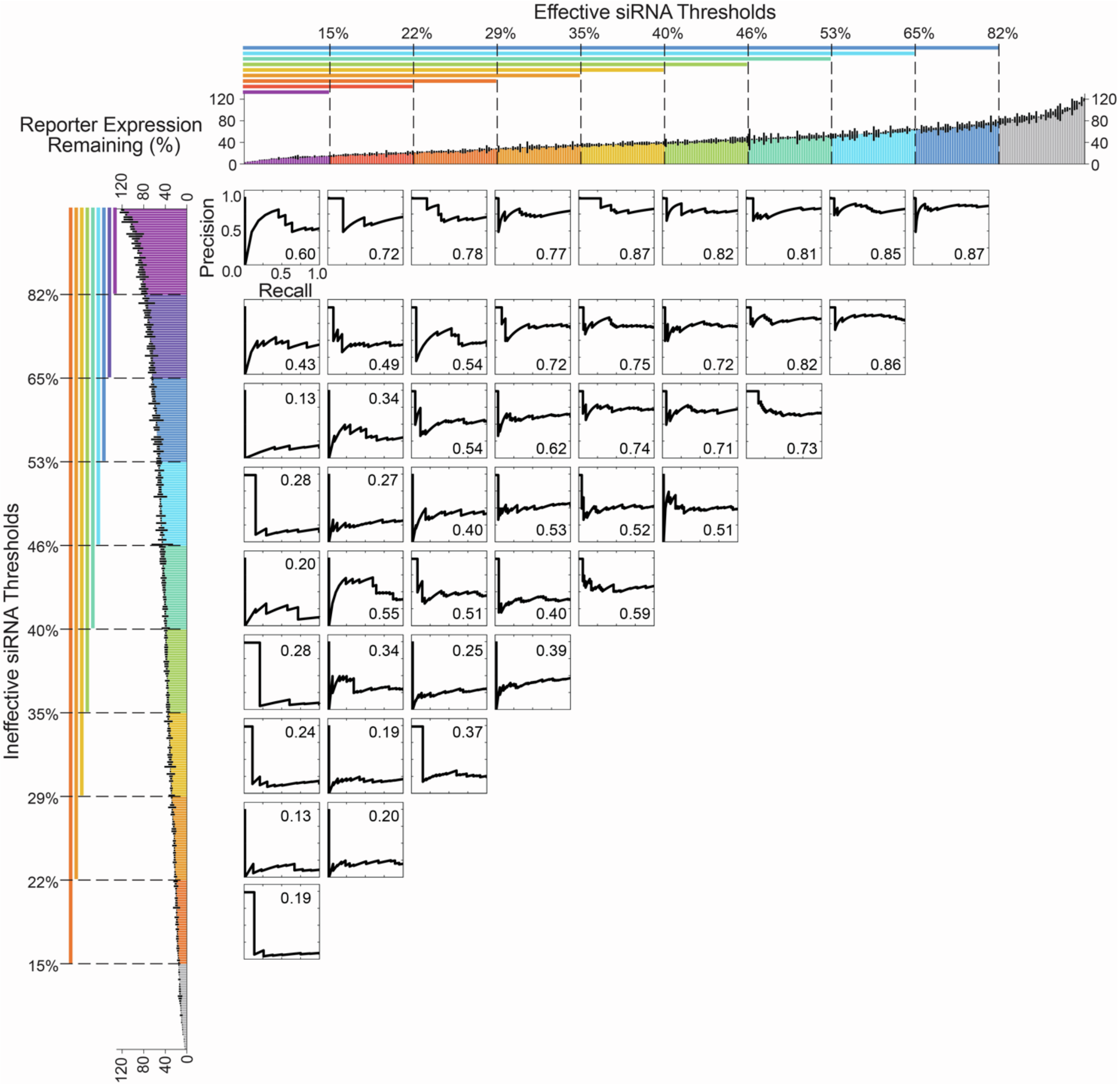

Figure S8. Model performance per classification threshold on randomized dataset, related to Figure 6. Precision-recall curves depicting performance of random forest models evaluated on a randomized holdout set. Area under the precision-recall curve values indicated in black on each curve. Bar plots at top and left depict all siRNA target expression data (as in Figure 2D) colored by effective (top) or ineffective (left) thresholds. Precision-recall curves are aligned to these bar plots to indicate the effective and ineffective thresholds used for training of the corresponding curve's model. Thresholds are inclusive of all data with expression values less than (for effective thresholds) or greater than (for ineffective thresholds) the threshold expression percentage.

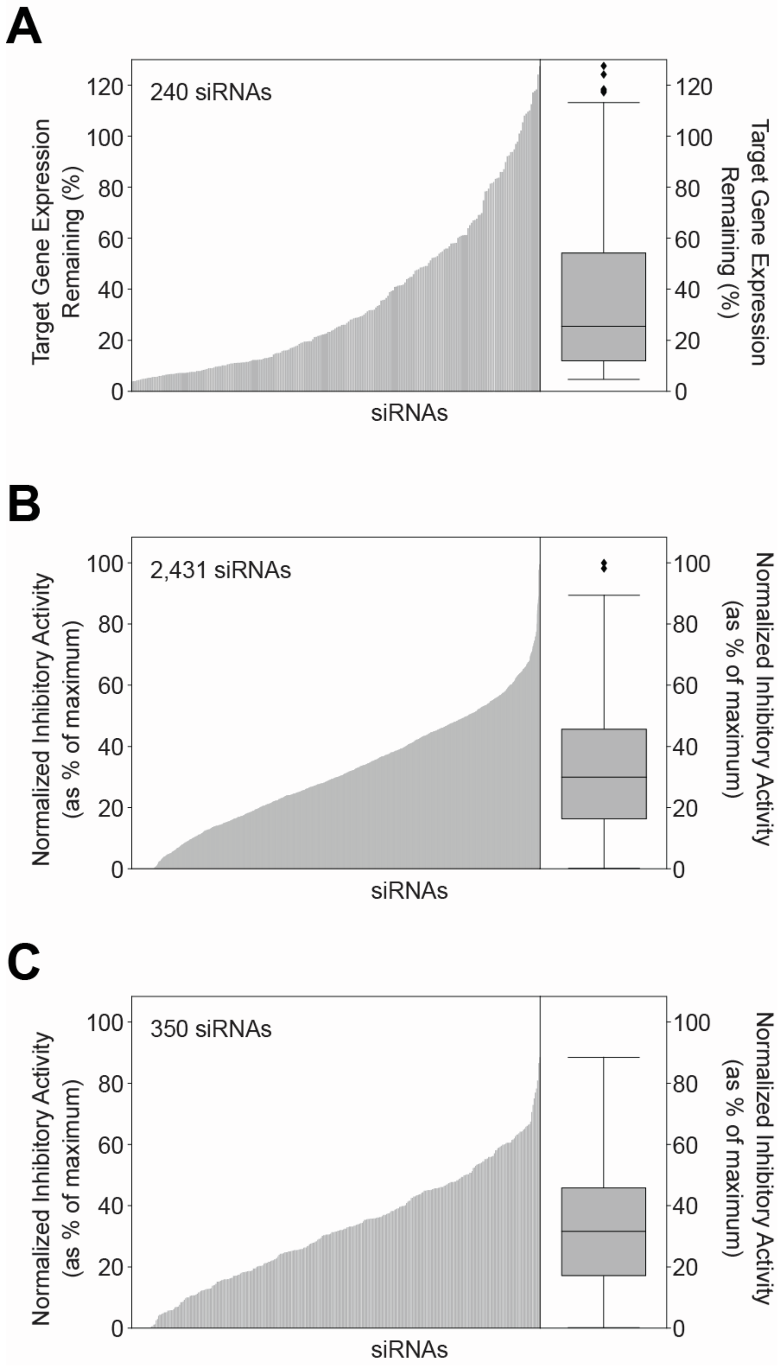

Figure S9. Distribution of siRNA efficacies of external datasets used for model building. (**A**) Gene silencing efficacy for external dataset of 240 nonmodified siRNAs evaluated in HEK293 cells by either branched-DNA assay or Luciferase reporter assay.[1] Each bar represents efficacy of a single siRNA sequence. Box and whisker plot depicts distribution of siRNA efficacies across the dataset. (**B**) Same as A but with gene silencing efficacy for external dataset of 2,431 nonmodified siRNAs evaluated in HeLa cells using a hypoxia-response element-Luciferase reporter assay.[2] (**C**) same as B but with subset of 350 randomly selected siRNAs used for model building.

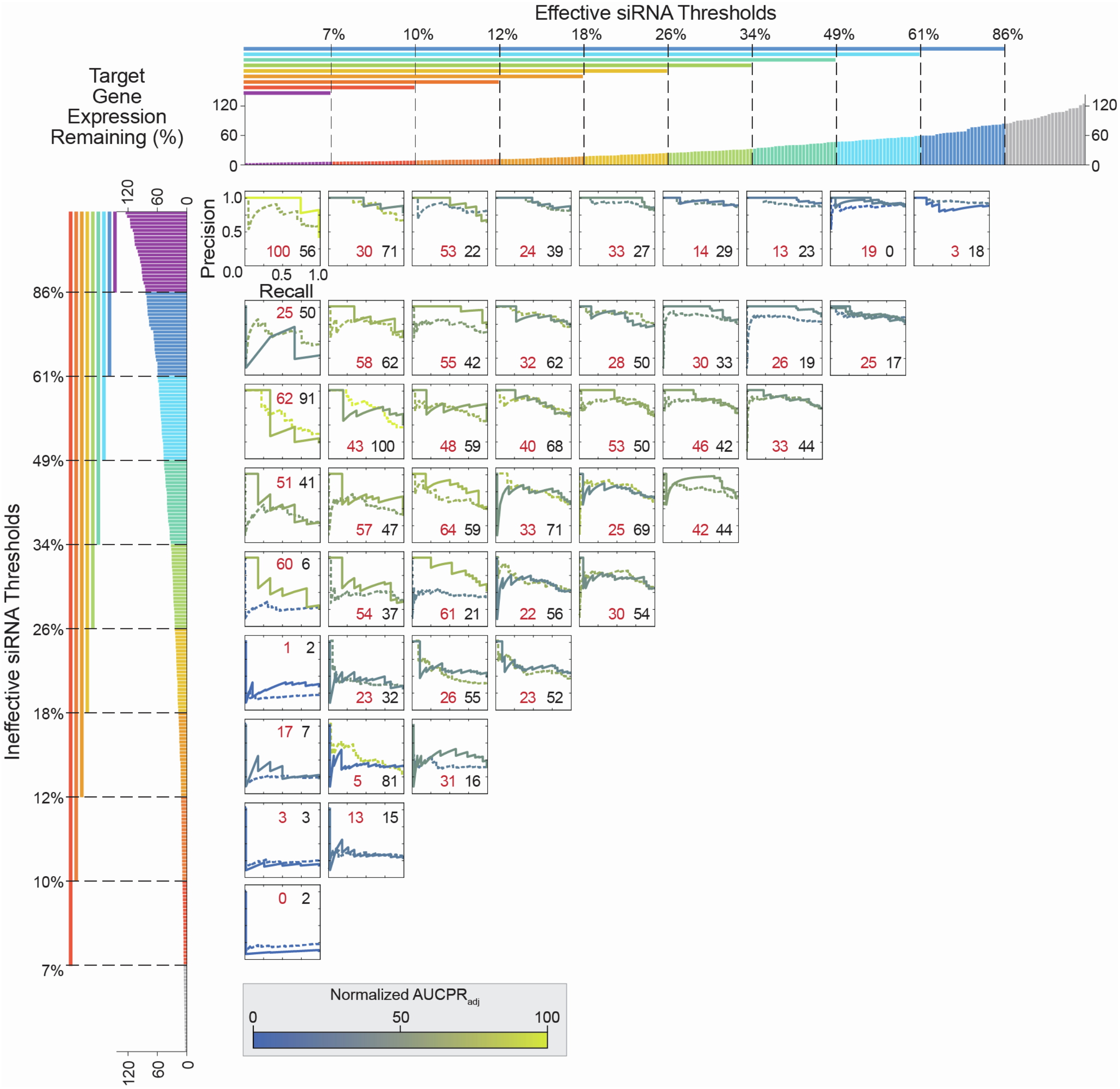

Figure S10. Model performance per classification threshold after applying model building framework on external siRNA dataset. Precision-recall curves depicting performance of random forest models built from a randomly subsetted external dataset of 240 nonmodified siRNAs.[1] Results are shown as in Figure 5: with evaluation on the holdout set (solid lines) and K-fold cross-validation (dotted lines), and colored by adjusted area under the precision-recall curve (AUCPR$_{adj}$, color bar). AUCPR$_{adj}$ values indicated at bottom right of each curve K-fold cross-validation (red) or holdout set evaluation (black). Bar plots at top and left depict siRNA normalized inhibitory activities expressed as a percentage, used in the model building framework colored by effective (top) or ineffective (left) thresholds. Precision-recall curves are aligned to these bar plots to indicate the effective and ineffective thresholds used for training of the corresponding curve's model. Thresholds are inclusive of all data with expression values less than (for effective thresholds) or greater than (for ineffective thresholds) the threshold expression percentage.

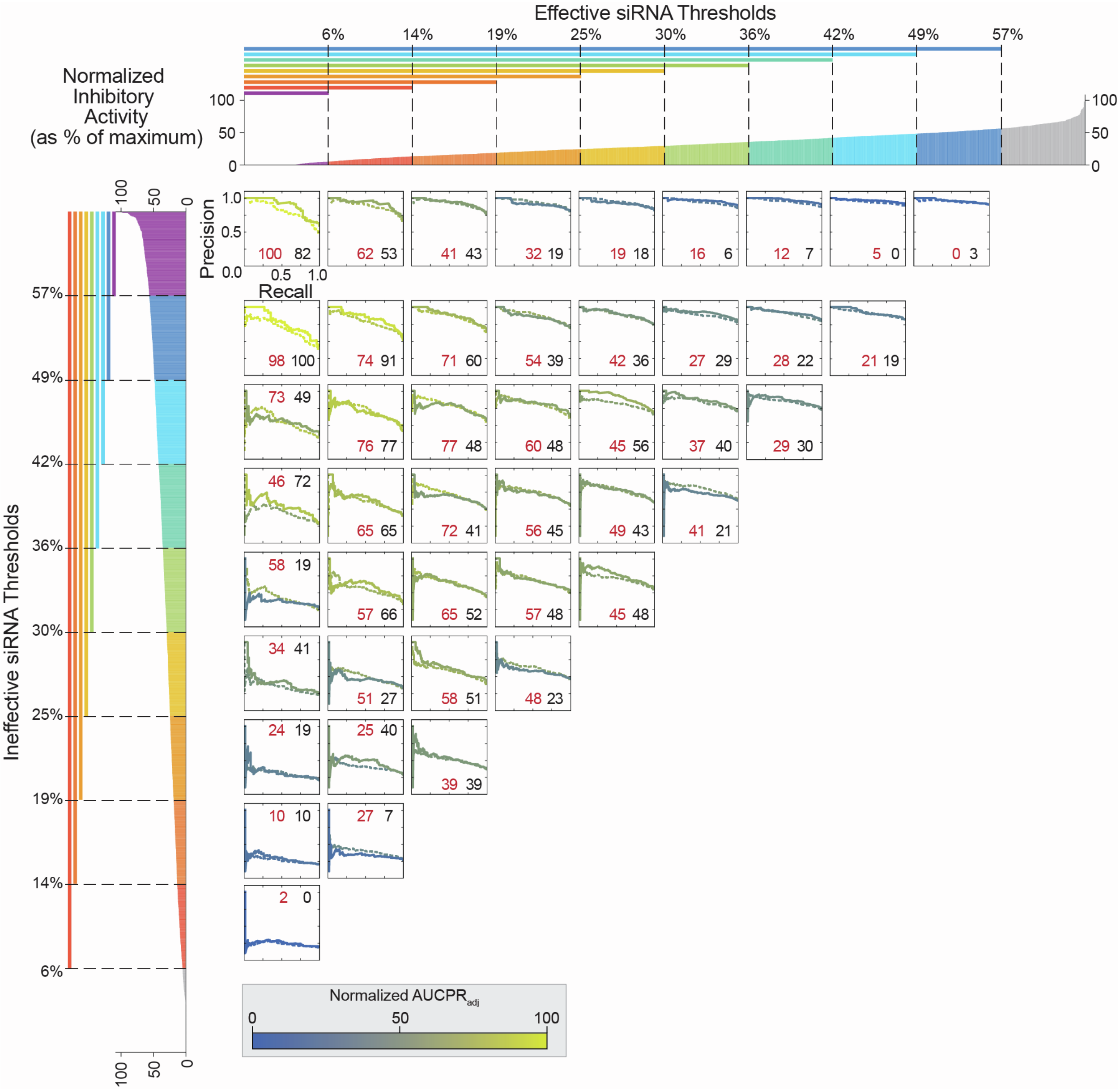

Figure S11. Model performance per classification threshold after applying model building framework on external siRNA dataset. Precision-recall curves depicting performance of random forest models built from an external dataset of 2,431 nonmodified siRNAs.[2] Results are shown as in Figure 5: with evaluation on the holdout set (solid lines) and K-fold cross-validation (dotted lines), and colored by adjusted area under the precision-recall curve ($AUCPR_{adj}$, color bar). $AUCPR_{adj}$ values indicated at bottom right of each curve K-fold cross-validation (red) or holdout set evaluation (black). Bar plots at top and left depict siRNA normalized inhibitory activities expressed as a percentage, used in the model building framework colored by effective (top) or ineffective (left) thresholds. Precision-recall curves are aligned to these bar plots to indicate the effective and ineffective thresholds used for training of the corresponding curve's model. Thresholds are inclusive of all data with expression values less than (for effective thresholds) or greater than (for ineffective thresholds) the threshold expression percentage.

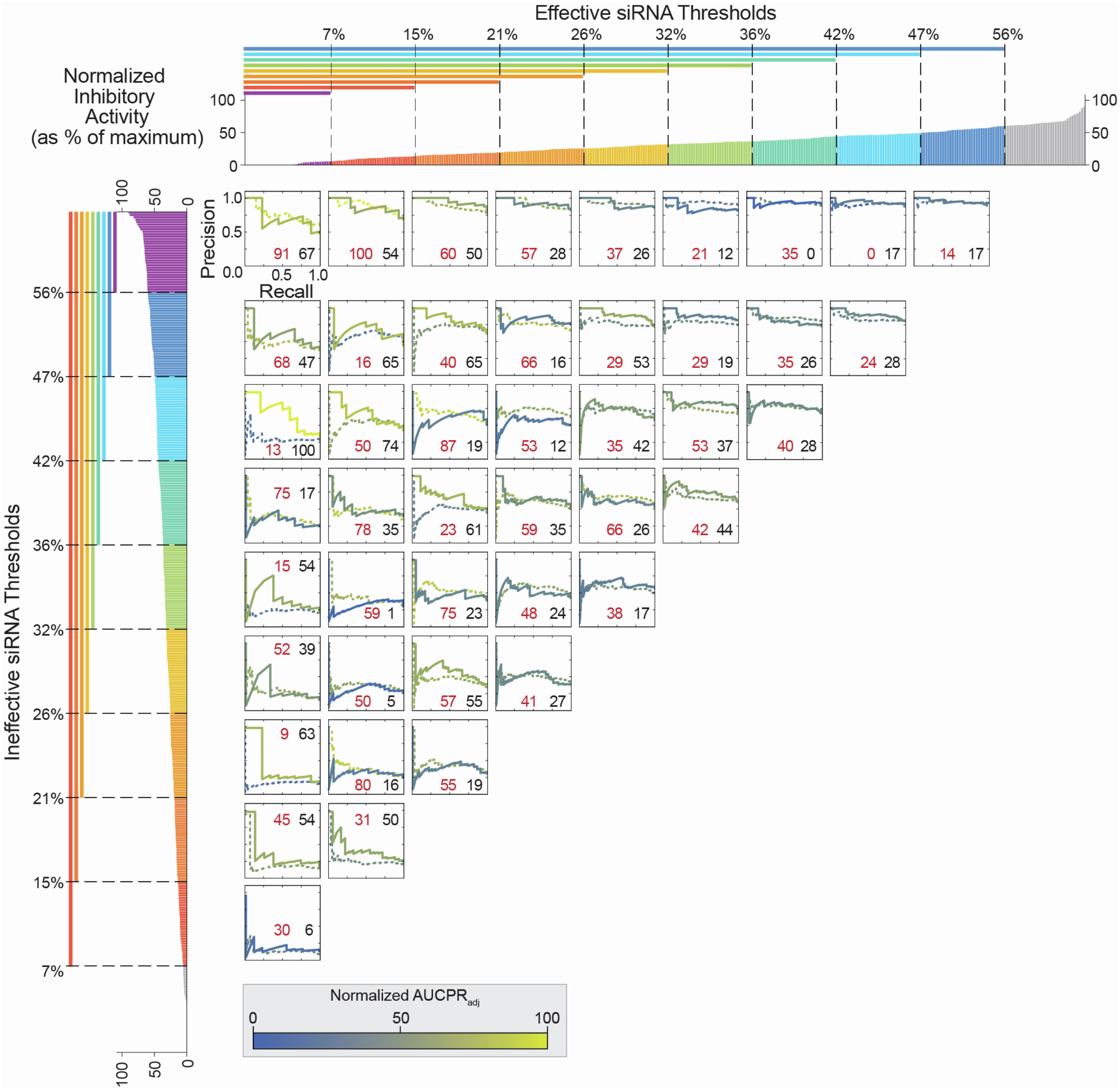

Figure S12. Model performance per classification threshold after applying model building framework on randomly downsized external siRNA dataset. Precision-recall curves depicting performance of random forest models built from a randomly subsetted external dataset of 350 nonmodified siRNAs.[2] Results are shown as in Figure 5: with evaluation on the holdout set (solid lines) and K-fold cross-validation (dotted lines), and colored by adjusted area under the precision-recall curve ($AUCPR_{adj}$, color bar). $AUCPR_{adj}$ values indicated at bottom right of each curve K-fold cross-validation (red) or holdout set evaluation (black). Bar plots at top and left depict subset of 350 randomly selected siRNA normalized inhibitory activities expressed as a percentage, used in the model building framework colored by effective (top) or ineffective (left) thresholds. Precision-recall curves are aligned to these bar plots to indicate the effective and ineffective thresholds used for training of the corresponding curve's model. Thresholds are inclusive of all data with expression values less than (for effective thresholds) or greater than (for ineffective thresholds) the threshold expression percentage.

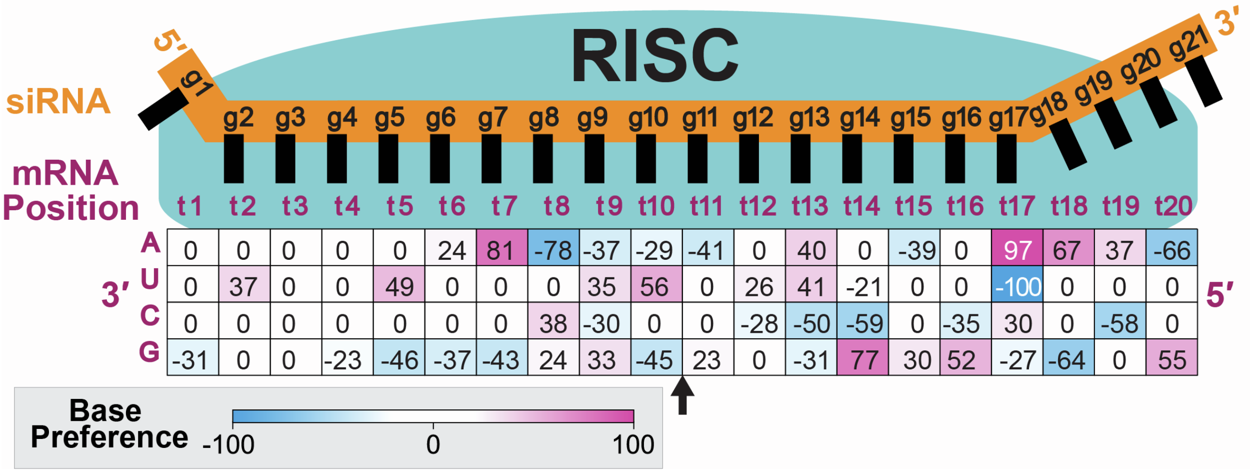

Figure S13. Target site base feature weights identified by siRNA efficacy prediction model method evaluated on external dataset. Base feature weights of the 20 nt target site extracted by proxy (see Results and Methods) from a random forest model developed from an external dataset 240 nonmodified 21 nt siRNAs.[1] Weights were extracted from the 20 nt target site sequence and are aligned with respect to the RNA-induced silencing complex (RISC) (see Figure 1A) in a matrix by nucleobase indicated in magenta along the left. Positions indicated for target (t) and guide (g) sequences. Weights are colored by value following the scale indicated. Magnitude indicates importance for the particular model, with higher magnitude weights indicating bases more important for prediction. Bases with zero weights are not important to prediction for the particular model. Direction (positive/negative) indicates favorability of a base with respect to identifying effective siRNAs for the particular model, with positive weights indicating a feature is favored in identifying effective siRNAs, and negative weights indicating disfavoring. Model developed using 10% effective and 61% ineffective thresholds respectively. Arrow indicates mRNA cleavage site between positions t10 and t11. Feature extraction performed at the 0.5 confidence margin.

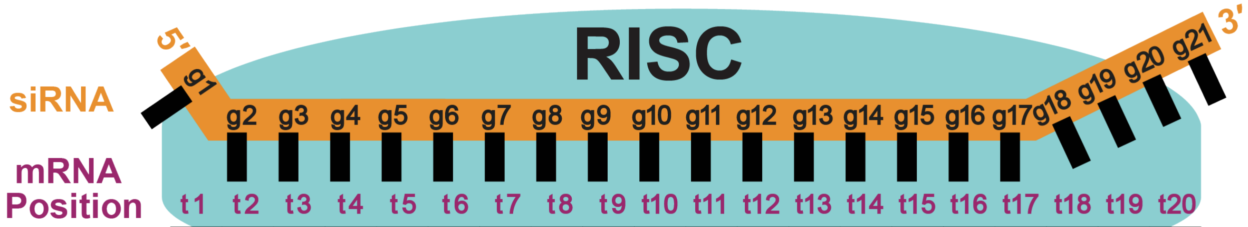

	t1	t2	t3	t4	t5	t6	t7	t8	t9	t10	t11	t12	t13	t14	t15	t16	t17	t18	t19	t20
A	0	0	50	40	0	0	30	30	-76	-30	0	40	28	0	-42	0	52	-100	-40	47
U	29	56	0	-38	-93	78	-50	-51	66	0	63	-29	-88	-22	0	-63	0	0	-23	-71
C	0	-53	-51	48	33	-96	57	48	-23	0	-24	41	0	43	0	0	-22	0	0	0
G	-24	0	21	-50	64	0	-36	-27	32	42	-59	-51	41	-40	40	57	-42	81	59	0

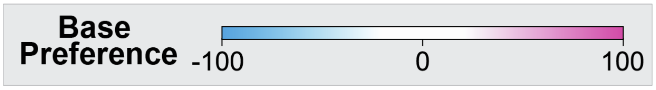

Figure S14. Target site base feature weights identified by siRNA efficacy prediction model method evaluated on external dataset. Base feature weights of the 20 nt target site extracted by proxy (see Results and Methods) from a random forest model developed from an external dataset of 2,431 nonmodified 21 nt siRNAs.[2] Weights were extracted from the 20 nt target site sequence and are aligned with respect to the RNA-induced silencing complex (RISC) (see Figure 1A) in a matrix by nucleobase indicated in magenta along the left. Positions indicated for target (t) and guide (g) sequences. Weights are colored by value following the scale indicated. Magnitude indicates importance for the particular model, with higher magnitude weights indicating bases more important for prediction. Bases with zero weights are not important to prediction for the particular model. Direction (positive/negative) indicates favorability of a base with respect to identifying effective siRNAs for the particular model, with positive weights indicating a feature is favored in identifying effective siRNAs, and negative weights indicating disfavoring. Model developed using 14% effective and 49% ineffective thresholds respectively. Arrow indicates mRNA cleavage site between positions t10 and t11. Feature extraction performed at the 0.5 confidence margin.

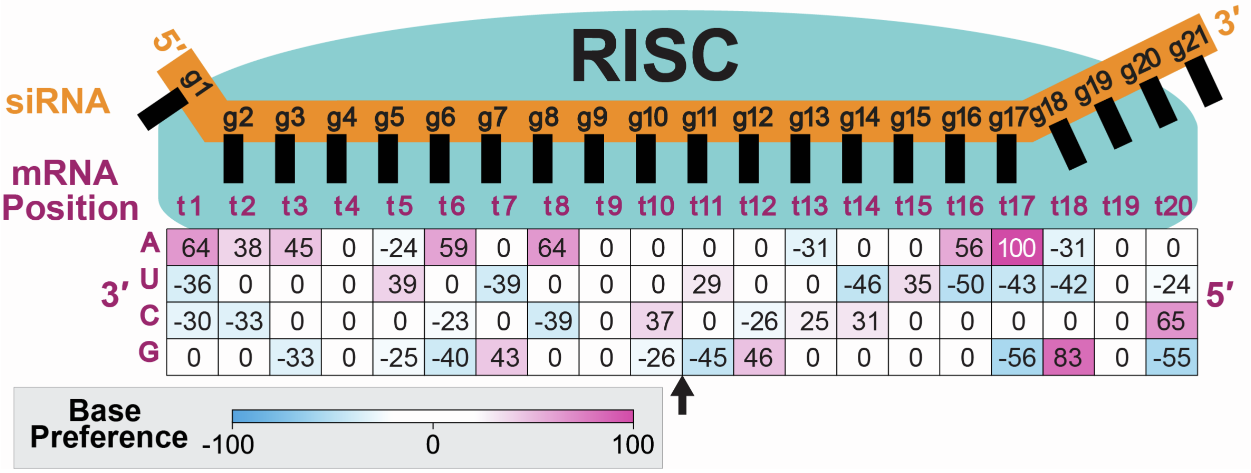

Figure S15. Target site base feature weights identified by siRNA efficacy prediction model method evaluated on randomly downsized external dataset. Base feature weights of the 20 nt target site extracted by proxy (see Results and Methods) from a random forest model developed from an external dataset of randomly subsetted 350 nonmodified 21 nt siRNAs.[2] Weights were extracted from the 20 nt target site sequence and are aligned with respect to the RNA-induced silencing complex (RISC) (see Figure 1A) in a matrix by nucleobase indicated in magenta along the left. Positions indicated for target (t) and guide (g) sequences. Weights are colored by value following the scale indicated. Magnitude indicates importance for the particular model, with higher magnitude weights indicating bases more important for prediction. Bases with zero weights are not important to prediction for the particular model. Direction (positive/negative) indicates favorability of a base with respect to identifying effective siRNAs for the particular model, with positive weights indicating a feature is favored in identifying effective siRNAs, and negative weights indicating disfavoring. Model developed using 15% effective and 42% ineffective thresholds respectively. Arrow indicates mRNA cleavage site between positions t10 and t11. Feature extraction performed at the 0.5 confidence margin.

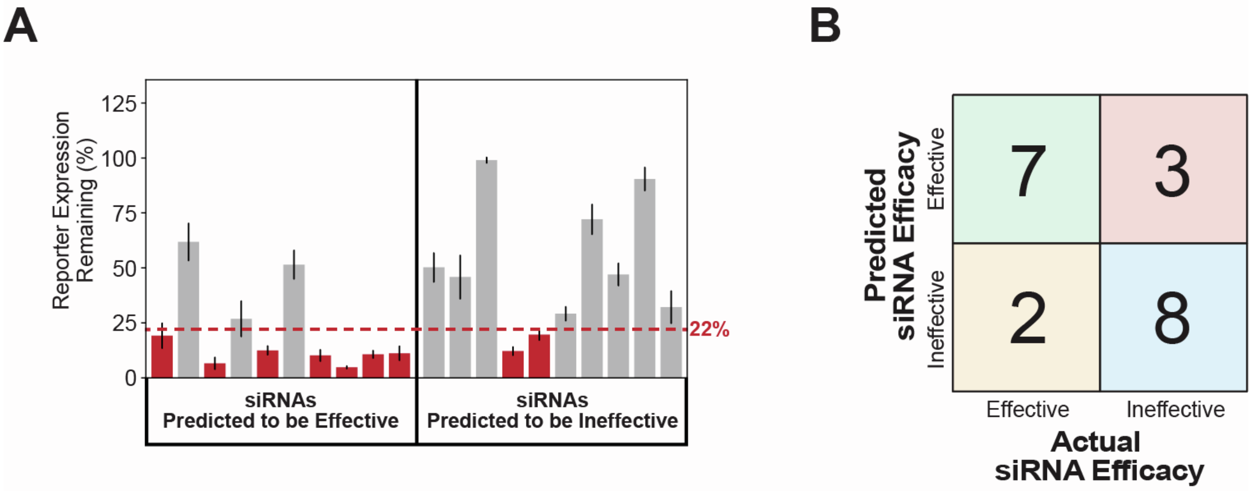

Figure S16. Experimental evaluation of siRNAs selected using random forest model. (**A**) Gene silencing efficacies for 20 modified siRNAs selected using the 22% effective and 53% ineffective threshold pair random forest model evaluated in HeLa cells using a dual Luciferase reporter assay (see Methods, Table S3). Each bar represents efficacy of a single siRNA sequence. siRNAs were selected by the model efficacy predictions as indicated at bottom: siRNAs with highest efficacy probability on the left, and siRNAs with highest inefficacy probability on the right. A 22% reporter expression remaining cutoff (red dotted line) is used to identify experimentally effective siRNAs (red bars). (**B**) Contingency table depicting classes predicted by the model and the actual classes after experimental evaluation using 22% reporter expression remaining threshold for classification. Contingency table constructed at the 0.5 confidence margin. Information regarding interpreting contingency tables can be found in Figures S2 and S3.

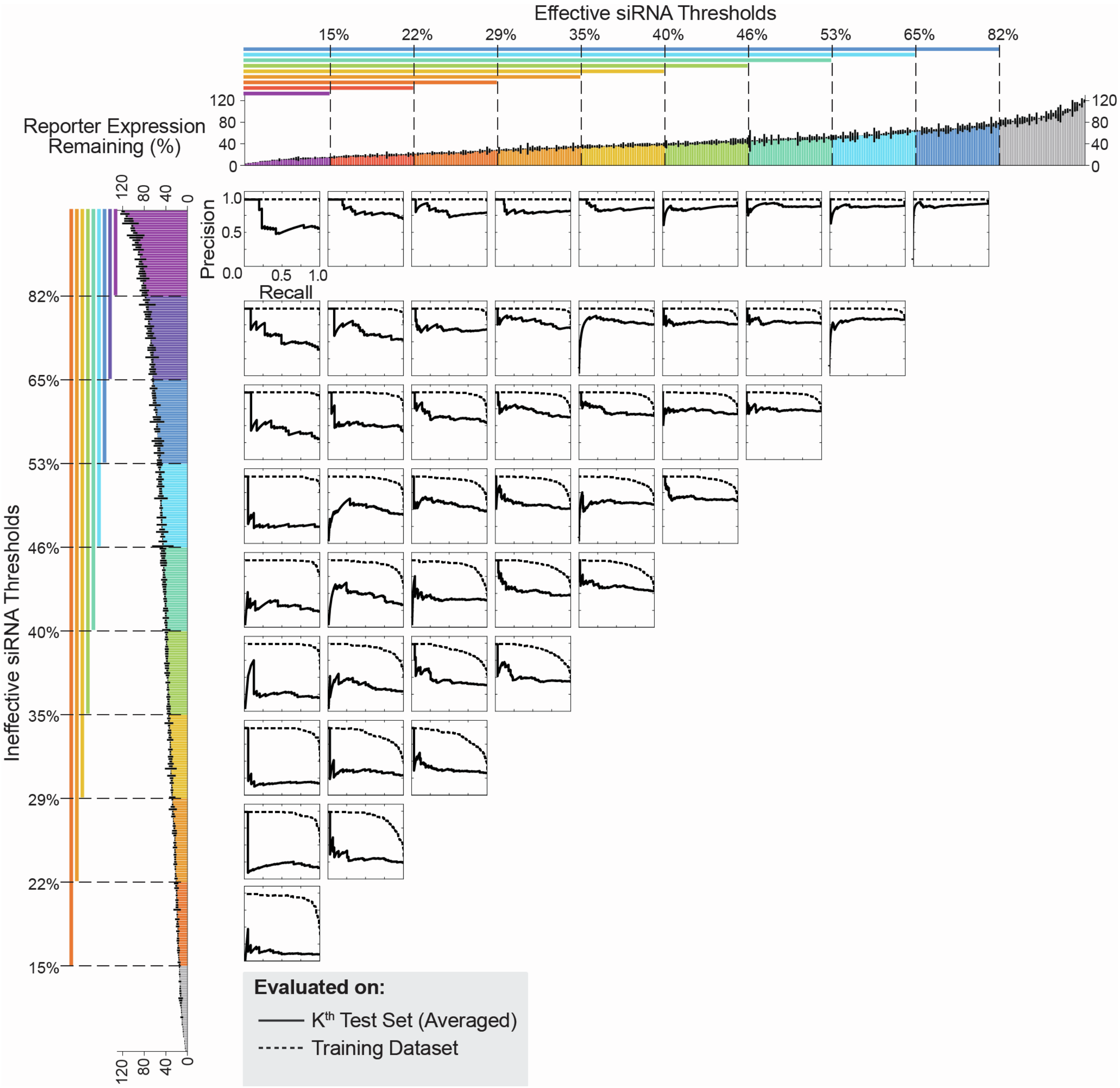

Figure S17. Comparing average model performances on training vs test sets during K-fold cross-validation, related to Figure 5. Precision recall curves for random forest classifiers evaluated on the training dataset (dotted curves) and the Kth test set (solid curves). Each set of overlaid curves represents a single random forest classifier trained with different effective and ineffective siRNA threshold combinations. Bar plots at top and left depict all siRNA target expression data (as in Figure 2D) colored by effective (top) or ineffective (left) thresholds. Curves are aligned to these bar plots to indicate the effective and ineffective thresholds used for training of that curve's classifier. Thresholds are inclusive of all data with expression values less than (for effective thresholds) or greater than (for ineffective thresholds) the threshold expression percentage. Grey bars indicate siRNAs excluded from model training for the indicated classification (effective or ineffective). Evaluations on each Kth subset were averaged over all K (K=10) rounds of cross-validation.

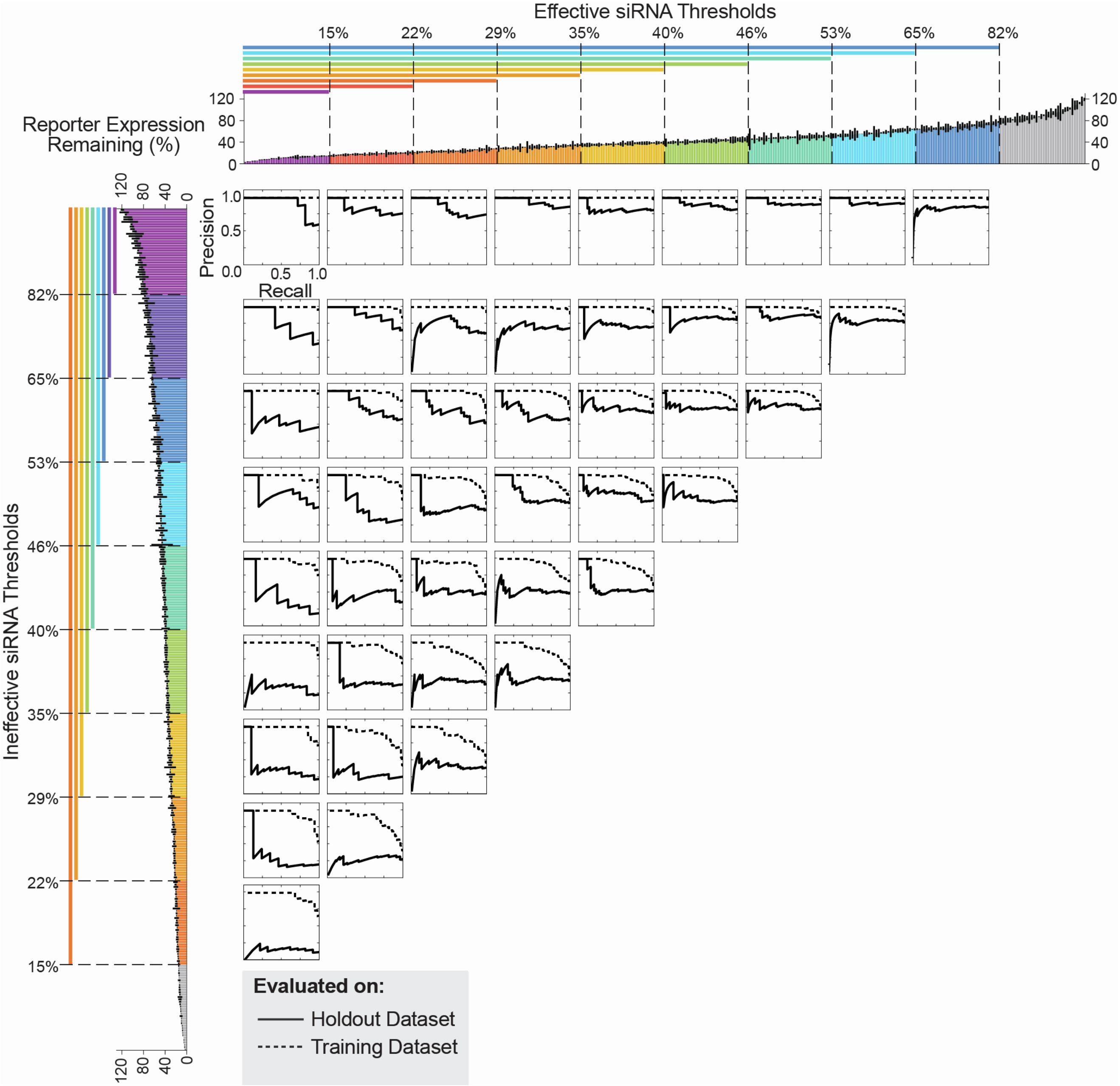

Figure S18. Comparing model performance on holdout dataset vs training dataset per classification threshold, related to Figure 5. Precision recall curves for random forest classifiers evaluated on the training dataset (dotted curves) and the holdout dataset (solid curves). Each set of overlaid curves represents a single random forest classifier trained with different effective and ineffective siRNA threshold combinations. Bar plots at top and left depict all siRNA target expression data (as in Figure 2D) colored by effective (top) or ineffective (left) thresholds. Curves are aligned to these bar plots to indicate the effective and ineffective thresholds used for training of that curve's classifier. Thresholds are inclusive of all data with expression values less than (for effective thresholds) or greater than (for ineffective thresholds) the threshold expression percentage. Grey bars indicate siRNAs excluded from model training for the indicated classification (effective or ineffective).

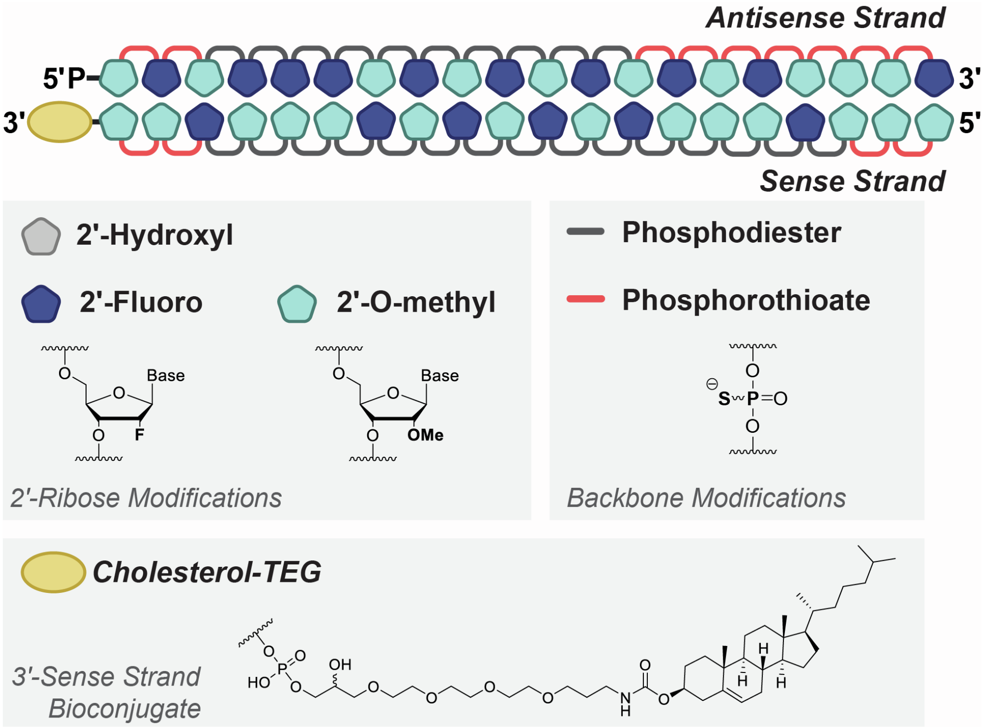

Figure S19. Chemical scaffold for fully modified siRNAs used for experimental evaluation of model. Chemical scaffold of blunt fully modified siRNAs consists of 20 nt sense and 20 nt antisense strands. Cholesterol was conjugated to the 3′ end of the sense strand. First antisense base from the 5′ end was fixed to 2′-O-methyl uridine. First seven 3′ and last two 5′ terminal antisense strand linkages were phosphorothioated. First and last two terminal sense strand linkages were phosphorothioated. On the sense strand, counting from 3′ to 5′ bases 3, 7, 9, 11, 13, and 17 were 2′-fluoro modified. On the antisense strand, counting from 5′ to 3′ bases 2, 4-6, 8, 10, 12, 14, 16, and 20 were 2′-fluoro modified. All other bases were 2′-O-methyl modified.

Table S1. Normalized adjusted area under the precision-recall curve per threshold pair evaluated on holdout set, related to Figure 5. Area under the precision-recall curve (AUCPR) and precision at recall equal to 1 ($P_{R=1}$) used to compute normalized adjusted AUCPR ($AUPCR_{adj}$). Each row represents a threshold pair that is defined by the specified effective and ineffective thresholds.

Normalized $AUCPR_{adj}$	Precision at Recall=1 ($P_{R=1}$)	AUCPR	Effective Threshold (% Reporter Expression Remaining)	Ineffective Threshold (% Reporter Expression Remaining)
100	0.588	0.904	80	15
99	0.435	0.750	65	15
84	0.149	0.420	40	15
74	0.645	0.873	65	22
72	0.302	0.530	46	22
67	0.564	0.863	52	22
66	0.500	0.704	46	15
64	0.510	0.708	52	29
52	0.182	0.345	29	15
48	0.565	0.707	46	35
42	0.552	0.672	52	35
41	0.158	0.283	22	15
40	0.603	0.718	46	40
39	0.327	0.442	40	22
37	0.743	0.844	80	29
34	0.351	0.449	35	22
34	0.595	0.691	65	29
33	0.190	0.287	35	15
31	0.440	0.526	52	15
31	0.823	0.905	80	46
28	0.760	0.832	80	22
28	0.217	0.297	29	22
28	0.449	0.526	40	29
28	0.864	0.933	80	35
28	0.667	0.740	52	40
27	0.500	0.571	40	40
24	0.455	0.519	46	29
22	0.596	0.650	46	46
22	0.719	0.773	52	52
17	0.738	0.774	52	46
16	0.354	0.390	35	29
16	0.901	0.929	80	52
16	0.850	0.881	65	52
15	0.695	0.724	65	40
14	0.119	0.151	15	15
14	0.463	0.492	40	35
14	0.913	0.933	80	65
13	0.360	0.388	29	29
13	0.803	0.824	65	46
12	0.409	0.432	35	35
11	0.811	0.825	80	40
9	0.764	0.773	65	65
3	0.673	0.660	65	35
1	0.241	0.230	22	22
0	0.854	0.828	80	80

Table S2. Normalized adjusted area under the precision-recall curve per threshold pair for K-fold cross-validation, related to Figure 5. Area under the precision-recall curve (AUCPR) and precision at recall equal to 1 ($P_{R=1}$) used to compute normalized adjusted AUCPR ($AUPCR_{adj}$). Each row represents a threshold pair that is defined by the specified effective and ineffective thresholds.

Normalized $AUCPR_{adj}$	Precision at Recall=1 ($P_{R=1}$)	AUCPR	Effective Threshold (% Reporter Expression Remaining)	Ineffective Threshold (% Reporter Expression Remaining)
100	0.365	0.573	15	65
83	0.277	0.434	15	52
73	0.515	0.625	22	65
72	0.701	0.828	22	80
64	0.306	0.447	22	40
57	0.395	0.563	22	52
57	0.525	0.636	29	52
57	0.457	0.572	29	46
57	0.696	0.794	35	65
54	0.617	0.715	35	52
48	0.551	0.640	15	80
46	0.360	0.461	29	35
43	0.638	0.712	40	52
42	0.454	0.539	35	40
40	0.403	0.489	22	46
38	0.521	0.593	40	40
37	0.502	0.574	35	46
37	0.413	0.492	35	35
35	0.262	0.348	22	35
33	0.205	0.291	22	22
33	0.617	0.673	46	46
32	0.299	0.375	29	29
30	0.200	0.280	15	40
28	0.755	0.789	40	65
28	0.664	0.706	46	52
27	0.162	0.240	15	35
24	0.261	0.325	22	29
24	0.758	0.784	46	65
22	0.798	0.817	29	80
22	0.774	0.796	52	65
21	0.711	0.735	52	52
20	0.892	0.900	52	80
18	0.125	0.188	15	22
17	0.823	0.830	35	80
16	0.677	0.694	29	65
14	0.215	0.263	15	46
14	0.873	0.871	40	80
14	0.559	0.581	40	46
12	0.373	0.405	29	40
7	0.135	0.174	15	29
7	0.904	0.884	65	80
3	0.831	0.810	65	65
2	0.104	0.136	15	15
2	0.936	0.904	80	80
0	0.897	0.864	46	80

Table S3. siRNAs selected by random forest model for experimental evaluation, related to Figures S16 and S19. Selected by model developed using 22% effective and 53% ineffective threshold pair applied to four human transcripts. Each row represents a single siRNA from the top ten highest confidence scores (predicted effective class) and bottom ten lowest confidence scores (predicted ineffective class). Silencing efficacies determined in HeLa cells by dual Luciferase reporter assay (see Methods) shown as the reporter expression remaining averaged across three independent experiments and expressed as a percentage of an untreated control with corresponding standard deviations.

Targeting Region	Target Transcript Gene	Predicted Class	Confidence Score	Reporter Expression Remaining (%)	Standard Deviation (%)
UUCAAUAUGCUAAAGAAGUA	*APP*	Effective	0.587	19.2	5.7
GUCCAAGUGUGGCUCAAAGG	*MAPT*	Effective	0.558	61.9	8.5
GGUCCUAAGCCCACAAUCAU	*MAPT*	Effective	0.553	6.6	2.7
UGAUCGGGCCCGAAAACGAA	*BACE1*	Effective	0.544	26.9	8.1
UUUUGAAAGGCUUUCCUCAG	*MAPT*	Effective	0.531	12.5	2.0
CUUUGUGAUUCCCUACCGCU	*APP*	Effective	0.530	51.6	6.5
CAUUGAGACUUCAAGCUUUU	*APP*	Effective	0.524	10.2	2.6
UAGUGCAUGAAUAGAUUCUC	*APP*	Effective	0.524	4.8	0.6
GUGGGAGUUCAGCUGCUUCU	*APP*	Effective	0.520	10.7	1.7
GUCACCUUAAAGGAGAUCAA	*SNCA*	Effective	0.513	11.2	3.2
UGCUGCCAUGAUUUUGGCCA	*MAPT*	Ineffective	0.299	50.3	6.6
AGCCUCUGAAGUUGGACAGC	*APP*	Ineffective	0.299	45.9	9.9
AUGGUUUCUGGCUAGGAGAG	*BACE1*	Ineffective	0.290	99.2	1.3
AUGAUCGCUUUCUACACUGU	*APP*	Ineffective	0.288	12.2	1.9
ACUUUCAGAACUGCUACCAU	*BACE1*	Ineffective	0.280	19.7	2.5
AUGGGUGCUGAAAAUAAACU	*SNCA*	Ineffective	0.277	29.2	3.2
AAGCAGCAUAUUUUAAAAAU	*SNCA*	Ineffective	0.275	72.2	6.8
CAAGUGACAAAUGUUGGAGG	*SNCA*	Ineffective	0.265	47.1	5.1
CAAAGUCCAGGCACAAGAGU	*MAPT*	Ineffective	0.263	90.6	5.3
AUUCUCCAAAACAAUUUUCU	*APP*	Ineffective	0.256	32.2	7.3

References

[1] Reynolds, A., Leake, D., Boese, Q., Scaringe, S., Marshall, W.S., and Khvorova, A. (2004). Rational siRNA design for RNA interference. Nat Biotechnol 22, 326–330.

[2] Huesken, D., Lange, J., Mickanin, C., Weiler, J., Asselbergs, F., Warner, J., Meloon, B., Engel, S., Rosenberg, A., Cohen, D., et al. (2005). Design of a genome-wide siRNA library using an artificial neural network. Nat Biotechnol *23*, 995–1001.
